# Supplementary material for: Integrating NMR and simulations reveals motions in the UUCG tetraloop
Source: Nucleic Acids Res. 2020 May 19;48(11):5839–48. doi: 10.1093/nar/gkaa399 (PMC7293013; doi:10.1093/nar/gkaa399)
Supplement: gkaa399_Supplemental_Files [file gkaa399_supplemental_files.zip › SI3_BME.html]

SI4\_BME


# Integrate simulations and experimental data¶

In this notebook we refine the MD simulation to match experimental data using the Bayesian/MaxEnt approach (BME). In brief, what we use as input is:

1. A set of experimental constraints ${F\_1^{exp} ... F\_m^{exp}}$ and associated errors ${\sigma\_1... \sigma\_m}$
2. The experimental observables back-calculated for each of the $n$ frames in the simulation ($F(x\_j)\_{i}^{calc}, i=1...m, j=1...n$)
3. The initial weights $w^0\_1...w^0\_n$. In a plain MD simulation the initial weights are uniform ($w\_i=1/n$), but we here use metadynamics, and the weights are given by $w^0\_j \propto \exp( V(x\_j)/kbt)$, where $V$is the final bias potential. The appendix at the end of this notebook describes how to calculate the bias using Plumed.

The output of the procedure is a new set of weights ${w\_1...w\_n}$ that are such that the new averages $<F\_i^{calc}> = \sum\_j^n w\_j F(x\_j)\_{i}^{calc} \approx F\_i^{exp} $. Clearly, the new weights can be used to calculate any desired quantity or distribution.

These steps are largely automatized by our BME script, that can be found here. In essence, the user has to perform the following actions:

```
# initialize the class, specifying the initial weights (if non-uniform)
rew = bme.Reweight(w0=bias,kbt=kbt)
# load the experimental and back-calculated data files
rew.load(exp,calc)
# Run the optimization. A regularization parameter, theta, has to be specified
chi2_before,chi2_after,effective_frames = rew.optimize(theta=t)
# Calculate new averages with the new weights. Note that the exp and calc can be the original 
data used as input (training set), or new data (cross-validation or test set)
# statistics are written to a file
chi = rew.weight_exp(exp,calc,out_filename)
```

In [2]:

```
import sys
import numpy as np
import os
# add BME to the path (should be changed and depends where the BME script is located)
sys.path.append('/home/sbottaro/Software/python_lib/BME/')
import bme_reweight as bme

# set the intial weights from metadynamics. The bias is passed along with kbt, so 
# that the initial weights are set proportional to exp(bias/kbt)
kbt = 0.008314462*280
bias = [float(line.split()[1]) for line in open("data/bias") if "#" not in line]

# this is a list of available data
lista = [["set_A","eNOE","eNOE_unidir","gn_eNOE"],\
        ["set_B","NOE","RDC","J3","CCRR"],\
        ["set_C","RDC1","RDC2"],\
        ["set_D","sPRE"]]


thetas =   [5,6,8,10,12.5,15,17.5,20,25,30,35,40,60,80,100,250,500,750,1000,2000]
thetas = thetas[::-1]

%mkdir -p data/theta_selection/set_A
%mkdir -p data/theta_selection/set_B
%mkdir -p data/theta_selection/set_C
%mkdir -p data/theta_selection/set_D
```

We choose the regularization parameter theta for each dataset by 10-fold cross-validation. First, we randomly select the 20% of the data from each dataset (which it will be used for training), while the rest is used for validation

In [3]:

```
# generate random row indeces that will be used later. 

train_fraction = 0.8
n_iterations = 5
columns = [[[] for j in range(len(lista[i])-1) ] for i in range(len(lista))]
for i in range(len(lista)):
    for j in range(1,len(lista[i])):
        for it in range(n_iterations):
            exp = "data/exp/%s/%s.exp.dat" % (lista[i][0],lista[i][j])
            calc = "data/calc/%s/%s.calc.dat" % (lista[i][0],lista[i][j])
            ll = sum([1 for line in open(exp) if "#" not in line])
            rr = np.arange(ll)
            np.random.shuffle(rr)
            columns[i][j-1].append(rr[:int(train_fraction*ll)])
```

In [2]:

```
import multiprocessing as mp
#pool = mp.Pool(mp.cpu_count())
print(mp.cpu_count())
pool = mp.Pool(40)

def calc(t):
    print(t,lista[i])
    for it in range(n_iterations):
        
        # initialize
        rew= bme.Reweight(w0=bias,kbt=kbt)
        
        for j in range(1,len(lista[i])):
            # define experimental and calculated datafile                                                                                                 
            exp = "data/exp/%s/%s.exp.dat" % (lista[i][0],lista[i][j])
            calc = "data/calc/%s/%s.calc.dat" % (lista[i][0],lista[i][j])
            # here we specify the columns (training examples that should be used)
            rew.load(exp,calc,cols=columns[i][j-1][it])
            
        # reweight
        before,after,neff = rew.optimize(theta=t)

        # calculate chi 
        for j in range(1,len(lista[i])):
            # define experimental and calculated datafile                                                                                                 
            exp = "data/exp/%s/%s.exp.dat" % (lista[i][0],lista[i][j])
            calc = "data/calc/%s/%s.calc.dat" % (lista[i][0],lista[i][j])
            ll = sum([1 for line in open(exp) if "#" not in line])
             # calculate chi on training set
            chi = rew.weight_exp(exp,calc,"data/theta_selection/%s/rw_%s_%s_theta_%d_%s" %\
                                     (lista[i][0],lista[i][0],lista[i][j],t,it),cols=columns[i][j-1][it])
            # calculate chi on validation set
            reverse = [k for k in range(ll) if k not in columns[i][j-1][it]]
            chi_r = rew.weight_exp(exp,calc,"data/theta_selection/%s/rw_%s_%s_theta_%d_%s_rev" %\
                                    (lista[i][0],lista[i][0],lista[i][j],t,it),cols=reverse)
        
        

results = []

# here we analyse only sPRE. Change the line below to analyse other datasets
#lista = [["set_B","NOE","RDC","J3","CCRR"]]
#for i in range(len(lista)):
    
#    results = [pool.apply(calc, args=(x,)) for x in thetas]
```

```
40
```

# $\theta$ selection¶

For each dataset, we run the optimization 10 times with a different set of training experimental data. We plot below the average training and validation $\chi^2$ for different values of $\theta$. A vertical, dashed line indicates the value of theta that is used for each dataset.

In [4]:

```
import glob
import numpy as np                                                                                                   

dd = ["set_A","set_B","set_C","set_D"]
nrep = 5

def calc_chi(mm):
    
    ll = glob.glob(mm)

    if(len(ll)==0):
        return np.nan, np.nan,0.0
    ntot = 0
    chi2_a_tot,chi2_b_tot = 0.,0.
    for el in ll:
        #print(el)
        fh = open(el)
        data = fh.readlines()
        nn = sum([1 for kk in data if "#" not in kk])
        chi2_b_tot += (float(data[-1].split()[-2])*nn)
        chi2_a_tot += (float(data[-1].split()[-1])*nn)
        ntot += nn
        neff=np.exp(float(data[0].split("=")[-1]))
    return chi2_b_tot/ntot, chi2_a_tot/ntot,neff

data = np.zeros((len(dd),len(thetas),nrep,5))

for i,d in enumerate(dd):
    for j,t in enumerate(thetas):
        for k in range(nrep):
            mm = "data/theta_selection/%s/rw_%s_*_theta_%d_%d.stats.dat" % (d,d,t,k)
            chi2_b_tot_train, chi2_a_tot_train,neff = calc_chi(mm)
            mm = "data/theta_selection/%s/rw_%s_*_theta_%d_%d_rev.stats.dat" % (d,d,t,k)
            chi2_b_tot_val, chi2_a_tot_val,neff1 = calc_chi(mm)

            data[i,j,k,0] = chi2_b_tot_train
            data[i,j,k,1] = chi2_a_tot_train
            data[i,j,k,2] = chi2_b_tot_val
            data[i,j,k,3] = chi2_a_tot_val
            data[i,j,k,4] = neff1
print(data.shape)

import matplotlib.pyplot as plt
theta_select = [20,40,60,500]

for i in range(data.shape[0]):
    for j in range(data.shape[2]):
        plt.plot(thetas,data[i,:,j,3],c='r',alpha=0.5,lw=0.5)
        plt.plot(thetas,data[i,:,j,1],c='0.5',alpha=0.5,lw=0.5)

    neff_mean = np.nanmean(data[i,:,:,4],axis=1)
    idx = np.abs(np.array(thetas) - theta_select[i]).argmin()
    #plt.plot(thetas,np.nanmean(data[i,:,:,4],axis=1),"o-",label="train",c='y')                                                                               
    print("Theta selected: % 4.1f" % theta_select[i])
    print("Effective fraction: % 4.1f" % (100*neff_mean[idx]))
    
    #for k in range(len(thetas)):
    #    print("%5.1f %5.3f %5.3f" % (thetas[k],neff_mean[k],np.nanmean(data[i,:,:,3],axis=1)[k]))
    plt.axvline(theta_select[i],c='k',ls='--')
    plt.plot(thetas,np.nanmean(data[i,:,:,1],axis=1),"o-",label="train",c='0.5',lw=2)
    plt.plot(thetas,np.nanmean(data[i,:,:,3],axis=1),"o-",label="validation",c='r',lw=2)
    plt.legend()
    plt.xscale('log')
    #plt.yscale('log')                                                                                                                                        
    #plt.xlim(0,500)                                                                                                                                          
    plt.savefig("plot_%d.png" % i)
    plt.xlabel(r"$\theta$")
    plt.title("MD+%s" % dd[i])
    plt.ylabel(r'$\chi^2$')
    if(i==3): plt.xlim(50,2000)
    plt.show()
    plt.close()
```

```
(4, 20, 5, 5)
Theta selected:  20.0
Effective fraction:  12.8
```

```
Theta selected:  40.0
Effective fraction:  10.4
```

```
Theta selected:  60.0
Effective fraction:  9.1
```

```
Theta selected:  500.0
Effective fraction:  9.2
```

```
/home/sbottaro/anaconda3/envs/py36/lib/python3.6/site-packages/ipykernel_launcher.py:60: RuntimeWarning: Mean of empty slice
/home/sbottaro/anaconda3/envs/py36/lib/python3.6/site-packages/ipykernel_launcher.py:61: RuntimeWarning: Mean of empty slice
```

# Repeat the reweighting with selected theta¶

In [5]:

```
%mkdir -p data/my_theta/set_A
%mkdir -p data/my_theta/set_B
%mkdir -p data/my_theta/set_C
%mkdir -p data/my_theta/set_D
```

In [6]:

```
# this is a list of available data
lista = [["set_A","eNOE","eNOE_unidir","gn_eNOE"],\
        ["set_B","NOE","RDC","J3","CCRR"],\
        ["set_C","RDC1","RDC2"],\
        ["set_D","sPRE"]]


nblocks = 4
bsize = int(len(bias)/nblocks)

theta_select = [20,40,60,500]


for i in range(len(lista)):
    
    for p in range(nblocks):
 
        start = p*bsize
        stop = start+bsize

        rew= bme.Reweight(w0=bias,kbt=kbt)
        for j in range(1,len(lista[i])):
            exp_c = "data/exp/%s/%s.exp.dat" % (lista[i][0],lista[i][j])
            calc_c = "data/calc/%s/%s.calc.dat" % (lista[i][0],lista[i][j])
            rew.load(exp_c,calc_c,rows=range(start,stop))
            
        # Optimize
        before,after,neff = rew.optimize(theta=theta_select[i])
            
        # print weights to file
        w_opt = rew.get_weights()
        w_0 = np.exp(np.array(bias)[range(start,stop)]/kbt)
        w_0 /= np.sum(w_0)
        stri = ""
        for ii in range(len(w_opt)):
            stri += "%10.4e %10.4e \n" % (w_0[ii],w_opt[ii])
        fhw = open("data/my_theta/weights_%s_t_%d_rep_%d.dat" % (lista[i][0],theta_select[i],p),"w")
        fhw.write(stri)
        fhw.close()
    
        # now loop over the other experimental data for cross-validation
        for l in range(len(lista)):
            for m in range(1,len(lista[l])):
                exp_c = "data/exp/%s/%s.exp.dat" % (lista[l][0],lista[l][m])
                calc_c = "data/calc/%s/%s.calc.dat" % (lista[l][0],lista[l][m])
                chi = rew.weight_exp(exp_c,calc_c,\
                                 "data/my_theta/%s/rw_%s_%s_theta_%d_rep_%d"\
                                  % (lista[i][0],lista[l][0],lista[l][m],theta_select[i],p),rows=range(start,stop))
                
    
    # do it again, to get weights wihtout bins - useful for 2d projections
    rew= bme.Reweight(w0=bias,kbt=kbt)
    for j in range(1,len(lista[i])):
        exp_c = "data/exp/%s/%s.exp.dat" % (lista[i][0],lista[i][j])
        calc_c = "data/calc/%s/%s.calc.dat" % (lista[i][0],lista[i][j])
        rew.load(exp_c,calc_c)
           
    # Optimize
    before,after,neff = rew.optimize(theta=theta_select[i])
    w_opt = rew.get_weights()
    w_0 = np.exp(np.array(bias)/kbt)
    w_0 /= np.sum(w_0)
    stri = ""
    for ii in range(len(w_opt)):
        stri += "%10.4e %10.4e \n" % (w_0[ii],w_opt[ii])
    fhw = open("data/my_theta/weights_%s_t_%d.dat" % (lista[i][0],theta_select[i]),"w")
    fhw.write(stri)
    fhw.close()
```

```
# Assuming weights given as minus free energies. w=exp(bias/kbt) kbt=  2.3280 
# Set non-uniform initial weights from file. Sum= 11503.331447473747 100001
# Warning: expt average         G2_H1;C13_H6=7.9748e-05 is larger than maximum value in simulation 4.0578e-05
# Warning: expt average        U11_H3;U11_H6=1.7960e-04 is larger than maximum value in simulation 1.0253e-04
# Warning: expt average         C5_H41;C5_H6=3.1401e-05 is smaller than minimum value in simulation 3.4807e-05
# Warning: expt average      C13_H1';C13_H3'=1.4759e-04 is smaller than minimum value in simulation 2.0084e-04
# Warning: expt average         C5_H4';C5_H6=5.0814e-04 is larger than maximum value in simulation 4.4971e-04
# Warning: expt average       G10_H4';G10_H8=5.0814e-04 is larger than maximum value in simulation 3.7553e-04
# Warning: expt average         C3_H1';C3_H6=2.2001e-04 is smaller than minimum value in simulation 2.5787e-04
# Warning: expt average        C3_H1';C3_H2'=8.5647e-04 is smaller than minimum value in simulation 9.7491e-04
# Warning: expt average        C3_H1';C3_H4'=2.8683e-04 is smaller than minimum value in simulation 3.3040e-04
# Warning: expt average         A4_H1';A4_H8=1.1375e-04 is smaller than minimum value in simulation 1.9556e-04
# Warning: expt average        A4_H1';A4_H2'=6.7424e-04 is smaller than minimum value in simulation 1.0877e-03
# Warning: expt average        A4_H1';A4_H3'=1.5557e-04 is smaller than minimum value in simulation 1.9359e-04
# Warning: expt average        A4_H1';A4_H4'=2.1300e-04 is smaller than minimum value in simulation 2.3085e-04
# Warning: expt average        U7_H1';U7_H2'=3.7857e-04 is smaller than minimum value in simulation 8.9415e-04
# Warning: expt average         C8_H1';C8_H6=2.0298e-04 is smaller than minimum value in simulation 2.4948e-04
# Warning: expt average        C8_H1';C8_H2'=4.5332e-04 is smaller than minimum value in simulation 8.6363e-04
# Warning: expt average        C8_H1';C8_H3'=1.1050e-04 is smaller than minimum value in simulation 1.8317e-04
# Warning: expt average      G12_H1';G12_H2'=9.6885e-04 is smaller than minimum value in simulation 1.0576e-03
# Warning: expt average      G12_H1';G12_H3'=9.5788e-05 is smaller than minimum value in simulation 2.0192e-04
# Warning: expt average      G12_H1';G12_H5"=3.3631e-05 is smaller than minimum value in simulation 3.4526e-05
# theta: 20
# Constrained optimization: Fall back to L-BFGS_B
# RDC scaling factors 7.3452e-01, 7.0530e-01
# RDC scaling factors 8.5915e-01, 8.3161e-01
# RDC scaling factors 1.1145e+00, 1.0232e+00
# RDC scaling factors 6.1902e-01, 5.9075e-01
# RDC scaling factors 6.1926e-03, 6.1804e-03
# Assuming weights given as minus free energies. w=exp(bias/kbt) kbt=  2.3280 
# Set non-uniform initial weights from file. Sum= 11503.331447473747 100001
# Warning: expt average         C5_H41;C5_H5=2.6328e-04 is smaller than minimum value in simulation 2.7802e-04
# Warning: expt average        U11_H3;U11_H6=1.7960e-04 is larger than maximum value in simulation 1.0536e-04
# Warning: expt average         C5_H41;C5_H6=3.1401e-05 is smaller than minimum value in simulation 3.4403e-05
# Warning: expt average      C13_H1';C13_H3'=1.4759e-04 is smaller than minimum value in simulation 1.7333e-04
# Warning: expt average        G2_H1';G2_H3'=1.9295e-04 is smaller than minimum value in simulation 2.0908e-04
# Warning: expt average         C5_H4';C5_H6=5.0814e-04 is larger than maximum value in simulation 4.3686e-04
# Warning: expt average       G10_H4';G10_H8=5.0814e-04 is larger than maximum value in simulation 3.3470e-04
# Warning: expt average         C3_H1';C3_H6=2.2001e-04 is smaller than minimum value in simulation 2.6356e-04
# Warning: expt average        C3_H1';C3_H2'=8.5647e-04 is smaller than minimum value in simulation 9.3465e-04
# Warning: expt average         A4_H1';A4_H8=1.1375e-04 is smaller than minimum value in simulation 2.0075e-04
# Warning: expt average        A4_H1';A4_H2'=6.7424e-04 is smaller than minimum value in simulation 9.5273e-04
# Warning: expt average        A4_H1';A4_H3'=1.5557e-04 is smaller than minimum value in simulation 1.6570e-04
# Warning: expt average        A4_H1';A4_H4'=2.1300e-04 is smaller than minimum value in simulation 2.2308e-04
# Warning: expt average        U7_H1';U7_H2'=3.7857e-04 is smaller than minimum value in simulation 8.1448e-04
# Warning: expt average         C8_H1';C8_H6=2.0298e-04 is smaller than minimum value in simulation 2.4195e-04
# Warning: expt average        C8_H1';C8_H2'=4.5332e-04 is smaller than minimum value in simulation 8.8685e-04
# Warning: expt average        C8_H1';C8_H3'=1.1050e-04 is smaller than minimum value in simulation 1.8286e-04
# Warning: expt average      G12_H1';G12_H3'=9.5788e-05 is smaller than minimum value in simulation 1.8902e-04
# theta: 20
# Constrained optimization: Fall back to L-BFGS_B
# RDC scaling factors 6.9143e-01, 7.2794e-01
# RDC scaling factors 8.4708e-01, 8.1926e-01
# RDC scaling factors 1.0045e+00, 1.0376e+00
# RDC scaling factors 6.1697e-01, 5.9303e-01
# RDC scaling factors 6.1923e-03, 6.1820e-03
# Assuming weights given as minus free energies. w=exp(bias/kbt) kbt=  2.3280 
# Set non-uniform initial weights from file. Sum= 11503.331447473747 100001
# Warning: expt average        U11_H3;U11_H6=1.7960e-04 is larger than maximum value in simulation 1.0162e-04
# Warning: expt average         C5_H41;C5_H6=3.1401e-05 is smaller than minimum value in simulation 3.5711e-05
# Warning: expt average      C13_H1';C13_H3'=1.4759e-04 is smaller than minimum value in simulation 1.8502e-04
# Warning: expt average        G2_H1';G2_H3'=1.9295e-04 is smaller than minimum value in simulation 1.9618e-04
# Warning: expt average         C5_H4';C5_H6=5.0814e-04 is larger than maximum value in simulation 4.4115e-04
# Warning: expt average       G10_H4';G10_H8=5.0814e-04 is larger than maximum value in simulation 3.3656e-04
# Warning: expt average         C3_H1';C3_H6=2.2001e-04 is smaller than minimum value in simulation 2.5194e-04
# Warning: expt average        C3_H1';C3_H2'=8.5647e-04 is smaller than minimum value in simulation 1.0128e-03
# Warning: expt average        C3_H1';C3_H4'=2.8683e-04 is smaller than minimum value in simulation 3.0865e-04
# Warning: expt average         A4_H1';A4_H8=1.1375e-04 is smaller than minimum value in simulation 1.9792e-04
# Warning: expt average        A4_H1';A4_H2'=6.7424e-04 is smaller than minimum value in simulation 9.9353e-04
# Warning: expt average        A4_H1';A4_H3'=1.5557e-04 is smaller than minimum value in simulation 1.9005e-04
# Warning: expt average        A4_H1';A4_H4'=2.1300e-04 is smaller than minimum value in simulation 2.7430e-04
# Warning: expt average        U7_H1';U7_H2'=3.7857e-04 is smaller than minimum value in simulation 8.9849e-04
# Warning: expt average         C8_H1';C8_H6=2.0298e-04 is smaller than minimum value in simulation 2.5141e-04
# Warning: expt average        C8_H1';C8_H2'=4.5332e-04 is smaller than minimum value in simulation 8.8274e-04
# Warning: expt average        C8_H1';C8_H3'=1.1050e-04 is smaller than minimum value in simulation 1.8587e-04
# Warning: expt average      G12_H1';G12_H3'=9.5788e-05 is smaller than minimum value in simulation 1.9118e-04
# Warning: expt average      G12_H1';G12_H5"=3.3631e-05 is smaller than minimum value in simulation 3.4571e-05
# theta: 20
# Constrained optimization: Fall back to L-BFGS_B
# RDC scaling factors 7.0720e-01, 6.8901e-01
# RDC scaling factors 8.4720e-01, 8.3981e-01
# RDC scaling factors 1.0635e+00, 1.0099e+00
# RDC scaling factors 6.6076e-01, 5.9522e-01
# RDC scaling factors 6.2253e-03, 6.1432e-03
# Assuming weights given as minus free energies. w=exp(bias/kbt) kbt=  2.3280 
# Set non-uniform initial weights from file. Sum= 11503.331447473747 100001
# Warning: expt average         C5_H41;C5_H5=2.6328e-04 is smaller than minimum value in simulation 2.6424e-04
# Warning: expt average        U11_H3;U11_H6=1.7960e-04 is larger than maximum value in simulation 1.0155e-04
# Warning: expt average         C5_H41;C5_H6=3.1401e-05 is smaller than minimum value in simulation 3.4777e-05
# Warning: expt average      C13_H1';C13_H3'=1.4759e-04 is smaller than minimum value in simulation 1.9801e-04
# Warning: expt average         C5_H4';C5_H6=5.0814e-04 is larger than maximum value in simulation 4.7732e-04
# Warning: expt average       G10_H4';G10_H8=5.0814e-04 is larger than maximum value in simulation 4.1958e-04
# Warning: expt average        G1_H3';G1_H5'=2.1300e-04 is smaller than minimum value in simulation 2.1332e-04
# Warning: expt average         C3_H1';C3_H6=2.2001e-04 is smaller than minimum value in simulation 2.5693e-04
# Warning: expt average        C3_H1';C3_H2'=8.5647e-04 is smaller than minimum value in simulation 8.9282e-04
# Warning: expt average        C3_H1';C3_H4'=2.8683e-04 is smaller than minimum value in simulation 2.8936e-04
# Warning: expt average         A4_H1';A4_H8=1.1375e-04 is smaller than minimum value in simulation 2.0058e-04
# Warning: expt average        A4_H1';A4_H2'=6.7424e-04 is smaller than minimum value in simulation 9.6466e-04
# Warning: expt average        A4_H1';A4_H3'=1.5557e-04 is smaller than minimum value in simulation 1.8249e-04
# Warning: expt average        A4_H1';A4_H4'=2.1300e-04 is smaller than minimum value in simulation 2.8829e-04
# Warning: expt average        U7_H1';U7_H2'=3.7857e-04 is smaller than minimum value in simulation 8.6651e-04
# Warning: expt average         C8_H1';C8_H6=2.0298e-04 is smaller than minimum value in simulation 2.4134e-04
# Warning: expt average        C8_H1';C8_H2'=4.5332e-04 is smaller than minimum value in simulation 8.5016e-04
# Warning: expt average        C8_H1';C8_H3'=1.1050e-04 is smaller than minimum value in simulation 1.8231e-04
# Warning: expt average      G12_H1';G12_H2'=9.6885e-04 is smaller than minimum value in simulation 1.0130e-03
# Warning: expt average      G12_H1';G12_H3'=9.5788e-05 is smaller than minimum value in simulation 1.9629e-04
# Warning: expt average      G12_H1';G12_H5"=3.3631e-05 is smaller than minimum value in simulation 3.4826e-05
# theta: 20
# Constrained optimization: Fall back to L-BFGS_B
# RDC scaling factors 6.5488e-01, 6.9189e-01
# RDC scaling factors 8.4150e-01, 8.3049e-01
# RDC scaling factors 1.0143e+00, 1.0162e+00
# RDC scaling factors 5.7163e-01, 5.6132e-01
# RDC scaling factors 6.1197e-03, 6.1848e-03
# Assuming weights given as minus free energies. w=exp(bias/kbt) kbt=  2.3280 
# Set non-uniform initial weights from file. Sum= 11503.331447473747 100001
# Warning: expt average        U11_H3;U11_H6=1.7960e-04 is larger than maximum value in simulation 1.0536e-04
# Warning: expt average         C5_H41;C5_H6=3.1401e-05 is smaller than minimum value in simulation 3.4403e-05
# Warning: expt average      C13_H1';C13_H3'=1.4759e-04 is smaller than minimum value in simulation 1.7333e-04
# Warning: expt average         C5_H4';C5_H6=5.0814e-04 is larger than maximum value in simulation 4.7732e-04
# Warning: expt average       G10_H4';G10_H8=5.0814e-04 is larger than maximum value in simulation 4.1958e-04
# Warning: expt average         C3_H1';C3_H6=2.2001e-04 is smaller than minimum value in simulation 2.5194e-04
# Warning: expt average        C3_H1';C3_H2'=8.5647e-04 is smaller than minimum value in simulation 8.9282e-04
# Warning: expt average         A4_H1';A4_H8=1.1375e-04 is smaller than minimum value in simulation 1.9556e-04
# Warning: expt average        A4_H1';A4_H2'=6.7424e-04 is smaller than minimum value in simulation 9.5273e-04
# Warning: expt average        A4_H1';A4_H3'=1.5557e-04 is smaller than minimum value in simulation 1.6570e-04
# Warning: expt average        A4_H1';A4_H4'=2.1300e-04 is smaller than minimum value in simulation 2.2308e-04
# Warning: expt average        U7_H1';U7_H2'=3.7857e-04 is smaller than minimum value in simulation 8.1448e-04
# Warning: expt average         C8_H1';C8_H6=2.0298e-04 is smaller than minimum value in simulation 2.4134e-04
# Warning: expt average        C8_H1';C8_H2'=4.5332e-04 is smaller than minimum value in simulation 8.5016e-04
# Warning: expt average        C8_H1';C8_H3'=1.1050e-04 is smaller than minimum value in simulation 1.8231e-04
# Warning: expt average      G12_H1';G12_H3'=9.5788e-05 is smaller than minimum value in simulation 1.8902e-04
# theta: 20
# Constrained optimization: Fall back to L-BFGS_B
# Assuming weights given as minus free energies. w=exp(bias/kbt) kbt=  2.3280 
# Set non-uniform initial weights from file. Sum= 11503.331447473747 100001
# Warning: expt average         G2_H1;C3_H42=4.1957e-03 is larger than maximum value in simulation 3.4104e-03
# Warning: expt average        G2_H1';G2_H2'=6.7551e-03 is larger than maximum value in simulation 6.1478e-03
# Warning: expt average        A4_H1';C5_H1'=1.2181e-03 is larger than maximum value in simulation 2.9391e-04
# Warning: expt average        A4_H1';A4_H3'=2.1202e-03 is larger than maximum value in simulation 6.5203e-04
# Warning: expt average        A4_H61;A4_H62=6.4132e-03 is smaller than minimum value in simulation 2.1540e-02
# Warning: expt average       C5_H3';C5_HO2'=1.0834e-04 is smaller than minimum value in simulation 1.5727e-04
# Warning: expt average        U6_H1';U6_H3'=6.2495e-04 is larger than maximum value in simulation 5.9501e-04
# Warning: expt average         U6_H5;U6_H3'=4.8305e-04 is larger than maximum value in simulation 4.0159e-04
# Warning: expt average        U6_H4';U6_H2'=7.3339e-04 is larger than maximum value in simulation 6.2990e-04
# Warning: expt average         C8_H5;C8_H2'=7.3339e-04 is larger than maximum value in simulation 3.1204e-04
# Warning: expt average       C8_H5';C8_H5''=1.3473e-02 is smaller than minimum value in simulation 1.8945e-02
# Warning: expt average      G10_H21;G10_H22=9.5773e-03 is smaller than minimum value in simulation 2.1926e-02
# Warning: expt average       G10_H8;G10_H4'=1.5485e-03 is larger than maximum value in simulation 3.7553e-04
# Warning: expt average    G10_H5'';G10_HO2'=9.8547e-04 is larger than maximum value in simulation 4.4159e-04
# Warning: expt average       U11_H1';U11_H3=2.2999e-04 is larger than maximum value in simulation 2.0509e-04
# Warning: expt average        U11_H3;U11_H6=2.2999e-04 is larger than maximum value in simulation 1.0253e-04
# Warning: expt average       G12_H1;G12_H21=3.1190e-04 is smaller than minimum value in simulation 3.8505e-04
# Warning: expt average      G12_H1';G12_H2'=9.1405e-04 is smaller than minimum value in simulation 1.0576e-03
# Warning: expt average      C13_H1';C13_H5'=4.2992e-04 is larger than maximum value in simulation 2.5794e-04
# Warning: expt average       C14_H1';C14_H6=8.4859e-04 is larger than maximum value in simulation 8.4516e-04
# RDC scaling factor 7.3452e-01
# Warning: expt average              11-1H5P=0.0000e+00 is smaller than minimum value in simulation 9.9199e-01
# Warning: expt average               3-2H5P=0.0000e+00 is smaller than minimum value in simulation 9.9199e-01
# RDC scaling factor 8.5915e-01
# Warning: expt average             U7:C4p-P=1.5100e+01 is larger than maximum value in simulation 1.5058e+01
# Warning: expt average             G2:C1-C2=-1.0100e+01 is smaller than minimum value in simulation -8.3810e+00
# Warning: expt average             C3:C1-C2=-8.7000e+00 is smaller than minimum value in simulation -8.3810e+00
# Warning: expt average             A4:C1-C2=-9.0000e+00 is smaller than minimum value in simulation -8.3810e+00
# Warning: expt average             C8:C1-C2=1.7300e+01 is larger than maximum value in simulation 1.6762e+01
# Warning: expt average            G12:C1-C2=-9.4000e+00 is smaller than minimum value in simulation -8.3810e+00
# Warning: expt average            G10:C3-C4=2.0700e+01 is larger than maximum value in simulation 1.6762e+01
# Warning: expt average             G9:C1-CC=1.7000e+01 is larger than maximum value in simulation 1.6132e+01
# theta: 40
# RDC scaling factors 7.3452e-01, 8.0822e-01
# RDC scaling factors 8.5915e-01, 8.8323e-01
# RDC scaling factors 1.1145e+00, 1.2271e+00
# RDC scaling factors 6.1902e-01, 8.8678e-01
# RDC scaling factors 6.1926e-03, 6.1662e-03
# Assuming weights given as minus free energies. w=exp(bias/kbt) kbt=  2.3280 
# Set non-uniform initial weights from file. Sum= 11503.331447473747 100001
# Warning: expt average         G2_H1;C3_H42=4.1957e-03 is larger than maximum value in simulation 3.7354e-03
# Warning: expt average        G2_H1';G2_H2'=6.7551e-03 is larger than maximum value in simulation 5.5398e-03
# Warning: expt average        A4_H1';C5_H1'=1.2181e-03 is larger than maximum value in simulation 1.7963e-04
# Warning: expt average        A4_H1';A4_H3'=2.1202e-03 is larger than maximum value in simulation 5.9781e-04
# Warning: expt average        A4_H61;A4_H62=6.4132e-03 is smaller than minimum value in simulation 2.2086e-02
# Warning: expt average       C5_H3';C5_HO2'=1.0834e-04 is smaller than minimum value in simulation 1.4964e-04
# Warning: expt average        U6_H4';U6_H2'=7.3339e-04 is larger than maximum value in simulation 6.9398e-04
# Warning: expt average         C8_H5;C8_H2'=7.3339e-04 is larger than maximum value in simulation 2.8834e-04
# Warning: expt average       C8_H5';C8_H5''=1.3473e-02 is smaller than minimum value in simulation 1.8295e-02
# Warning: expt average         C5_H5;G10_H1=4.4437e-04 is larger than maximum value in simulation 1.2434e-04
# Warning: expt average      G10_H21;G10_H22=9.5773e-03 is smaller than minimum value in simulation 2.2985e-02
# Warning: expt average       G10_H8;G10_H4'=1.5485e-03 is larger than maximum value in simulation 3.3470e-04
# Warning: expt average    G10_H5'';G10_HO2'=9.8547e-04 is larger than maximum value in simulation 3.8937e-04
# Warning: expt average       U11_H1';U11_H3=2.2999e-04 is larger than maximum value in simulation 2.0671e-04
# Warning: expt average        U11_H3;U11_H6=2.2999e-04 is larger than maximum value in simulation 1.0536e-04
# Warning: expt average       G12_H1;G12_H21=3.1190e-04 is smaller than minimum value in simulation 3.9446e-04
# Warning: expt average      G12_H1';G12_H2'=9.1405e-04 is smaller than minimum value in simulation 9.5220e-04
# Warning: expt average      C13_H1';C13_H5'=4.2992e-04 is larger than maximum value in simulation 2.3811e-04
# Warning: expt average      C13_H1';C13_H3'=6.0346e-04 is larger than maximum value in simulation 5.8506e-04
# RDC scaling factor 6.9143e-01
# Warning: expt average              11-1H5P=0.0000e+00 is smaller than minimum value in simulation 9.9199e-01
# Warning: expt average               3-2H5P=0.0000e+00 is smaller than minimum value in simulation 9.9199e-01
# RDC scaling factor 8.4708e-01
# Warning: expt average             U7:C4p-P=1.5100e+01 is larger than maximum value in simulation 1.4843e+01
# Warning: expt average             G2:C1-C2=-1.0100e+01 is smaller than minimum value in simulation -8.2632e+00
# Warning: expt average             C3:C1-C2=-8.7000e+00 is smaller than minimum value in simulation -8.2632e+00
# Warning: expt average             A4:C1-C2=-9.0000e+00 is smaller than minimum value in simulation -8.2632e+00
# Warning: expt average             C8:C1-C2=1.7300e+01 is larger than maximum value in simulation 1.6527e+01
# Warning: expt average             G9:C1-C2=-8.3000e+00 is smaller than minimum value in simulation -8.2632e+00
# Warning: expt average            G12:C1-C2=-9.4000e+00 is smaller than minimum value in simulation -8.2632e+00
# Warning: expt average            G10:C3-C4=2.0700e+01 is larger than maximum value in simulation 1.6527e+01
# Warning: expt average             G9:C1-CC=1.7000e+01 is larger than maximum value in simulation 1.5906e+01
# theta: 40
# RDC scaling factors 6.9143e-01, 8.2608e-01
# RDC scaling factors 8.4708e-01, 8.8542e-01
# RDC scaling factors 1.0045e+00, 1.2807e+00
# RDC scaling factors 6.1697e-01, 1.0003e+00
# RDC scaling factors 6.1923e-03, 6.1426e-03
# Assuming weights given as minus free energies. w=exp(bias/kbt) kbt=  2.3280 
# Set non-uniform initial weights from file. Sum= 11503.331447473747 100001
# Warning: expt average         G2_H1;C3_H42=4.1957e-03 is larger than maximum value in simulation 3.4601e-03
# Warning: expt average        G2_H1';G2_H2'=6.7551e-03 is larger than maximum value in simulation 5.1614e-03
# Warning: expt average         G2_H8;G2_H5'=1.1332e-02 is larger than maximum value in simulation 9.8437e-03
# Warning: expt average        A4_H1';C5_H1'=1.2181e-03 is larger than maximum value in simulation 3.1302e-04
# Warning: expt average        A4_H1';A4_H3'=2.1202e-03 is larger than maximum value in simulation 6.0515e-04
# Warning: expt average        A4_H61;A4_H62=6.4132e-03 is smaller than minimum value in simulation 2.1697e-02
# Warning: expt average       C5_H3';C5_HO2'=1.0834e-04 is smaller than minimum value in simulation 1.4686e-04
# Warning: expt average         U6_H5;U6_H3'=4.8305e-04 is larger than maximum value in simulation 3.9453e-04
# Warning: expt average        U6_H4';U6_H2'=7.3339e-04 is larger than maximum value in simulation 6.9958e-04
# Warning: expt average         C8_H5;C8_H2'=7.3339e-04 is larger than maximum value in simulation 3.1642e-04
# Warning: expt average       C8_H5';C8_H5''=1.3473e-02 is smaller than minimum value in simulation 1.9567e-02
# Warning: expt average        G9_H1';G9_H2'=5.3655e-03 is larger than maximum value in simulation 5.3373e-03
# Warning: expt average         C5_H5;G10_H1=4.4437e-04 is larger than maximum value in simulation 1.2044e-04
# Warning: expt average      G10_H21;G10_H22=9.5773e-03 is smaller than minimum value in simulation 2.2685e-02
# Warning: expt average       G10_H8;G10_H4'=1.5485e-03 is larger than maximum value in simulation 3.3656e-04
# Warning: expt average    G10_H5'';G10_HO2'=9.8547e-04 is larger than maximum value in simulation 3.6487e-04
# Warning: expt average      U11_H1';U11_H2'=4.9786e-03 is larger than maximum value in simulation 4.9626e-03
# Warning: expt average       U11_H1';U11_H3=2.2999e-04 is larger than maximum value in simulation 2.1136e-04
# Warning: expt average        U11_H3;U11_H6=2.2999e-04 is larger than maximum value in simulation 1.0162e-04
# Warning: expt average       G12_H1;G12_H21=3.1190e-04 is smaller than minimum value in simulation 3.6376e-04
# Warning: expt average      G12_H1';G12_H2'=9.1405e-04 is smaller than minimum value in simulation 9.2853e-04
# Warning: expt average      C13_H1';C13_H5'=4.2992e-04 is larger than maximum value in simulation 3.0289e-04
# RDC scaling factor 7.0720e-01
# Warning: expt average              11-1H5P=0.0000e+00 is smaller than minimum value in simulation 9.9200e-01
# Warning: expt average               3-2H5P=0.0000e+00 is smaller than minimum value in simulation 9.9199e-01
# RDC scaling factor 8.4720e-01
# Warning: expt average             U7:C4p-P=1.5100e+01 is larger than maximum value in simulation 1.4853e+01
# Warning: expt average             G2:C1-C2=-1.0100e+01 is smaller than minimum value in simulation -8.2643e+00
# Warning: expt average             C3:C1-C2=-8.7000e+00 is smaller than minimum value in simulation -8.2643e+00
# Warning: expt average             A4:C1-C2=-9.0000e+00 is smaller than minimum value in simulation -8.2643e+00
# Warning: expt average             C8:C1-C2=1.7300e+01 is larger than maximum value in simulation 1.6527e+01
# Warning: expt average             G9:C1-C2=-8.3000e+00 is smaller than minimum value in simulation -8.2643e+00
# Warning: expt average            G12:C1-C2=-9.4000e+00 is smaller than minimum value in simulation -8.2643e+00
# Warning: expt average            G10:C3-C4=2.0700e+01 is larger than maximum value in simulation 1.6529e+01
# Warning: expt average             G9:C1-CC=1.7000e+01 is larger than maximum value in simulation 1.5907e+01
# theta: 40
# RDC scaling factors 7.0720e-01, 8.2259e-01
# RDC scaling factors 8.4720e-01, 8.7700e-01
# RDC scaling factors 1.0635e+00, 1.2920e+00
# RDC scaling factors 6.6076e-01, 9.8834e-01
# RDC scaling factors 6.2253e-03, 6.1109e-03
# Assuming weights given as minus free energies. w=exp(bias/kbt) kbt=  2.3280 
# Set non-uniform initial weights from file. Sum= 11503.331447473747 100001
# Warning: expt average         G2_H1;C3_H42=4.1957e-03 is larger than maximum value in simulation 2.9404e-03
# Warning: expt average        G2_H1';G2_H2'=6.7551e-03 is larger than maximum value in simulation 5.3548e-03
# Warning: expt average         G2_H8;G2_H5'=1.1332e-02 is larger than maximum value in simulation 5.8487e-03
# Warning: expt average        A4_H1';C5_H1'=1.2181e-03 is larger than maximum value in simulation 1.6282e-04
# Warning: expt average        A4_H1';A4_H3'=2.1202e-03 is larger than maximum value in simulation 6.9933e-04
# Warning: expt average        A4_H61;A4_H62=6.4132e-03 is smaller than minimum value in simulation 2.2136e-02
# Warning: expt average       C5_H3';C5_HO2'=1.0834e-04 is smaller than minimum value in simulation 1.5321e-04
# Warning: expt average        U6_H4';U6_H2'=7.3339e-04 is larger than maximum value in simulation 6.8050e-04
# Warning: expt average         C8_H5;C8_H2'=7.3339e-04 is larger than maximum value in simulation 3.2350e-04
# Warning: expt average       C8_H5';C8_H5''=1.3473e-02 is smaller than minimum value in simulation 1.6214e-02
# Warning: expt average        G9_H1';G9_H2'=5.3655e-03 is larger than maximum value in simulation 4.7329e-03
# Warning: expt average         C5_H5;G10_H1=4.4437e-04 is larger than maximum value in simulation 1.5334e-04
# Warning: expt average      G10_H21;G10_H22=9.5773e-03 is smaller than minimum value in simulation 2.2426e-02
# Warning: expt average       G10_H8;G10_H4'=1.5485e-03 is larger than maximum value in simulation 4.1958e-04
# Warning: expt average    G10_H5'';G10_HO2'=9.8547e-04 is larger than maximum value in simulation 5.7105e-04
# Warning: expt average      U11_H1';U11_H2'=4.9786e-03 is larger than maximum value in simulation 4.7844e-03
# Warning: expt average       U11_H1';U11_H3=2.2999e-04 is larger than maximum value in simulation 2.0134e-04
# Warning: expt average        U11_H3;U11_H6=2.2999e-04 is larger than maximum value in simulation 1.0155e-04
# Warning: expt average       G12_H1;G12_H21=3.1190e-04 is smaller than minimum value in simulation 3.7625e-04
# Warning: expt average      G12_H1';U11_H2'=1.0841e-03 is larger than maximum value in simulation 8.6909e-04
# Warning: expt average      G12_H1';G12_H2'=9.1405e-04 is smaller than minimum value in simulation 1.0130e-03
# Warning: expt average      C13_H1';C13_H5'=4.2992e-04 is larger than maximum value in simulation 2.5346e-04
# Warning: expt average       C13_H1';C13_H6=1.0043e-03 is larger than maximum value in simulation 8.9282e-04
# RDC scaling factor 6.5488e-01
# Warning: expt average              11-1H5P=0.0000e+00 is smaller than minimum value in simulation 9.9199e-01
# Warning: expt average               3-2H5P=0.0000e+00 is smaller than minimum value in simulation 9.9199e-01
# RDC scaling factor 8.4150e-01
# Warning: expt average             U7:C4p-P=1.5100e+01 is larger than maximum value in simulation 1.4752e+01
# Warning: expt average             G2:C1-C2=-1.0100e+01 is smaller than minimum value in simulation -8.2087e+00
# Warning: expt average             C3:C1-C2=-8.7000e+00 is smaller than minimum value in simulation -8.2087e+00
# Warning: expt average             A4:C1-C2=-9.0000e+00 is smaller than minimum value in simulation -8.2087e+00
# Warning: expt average             C8:C1-C2=1.7300e+01 is larger than maximum value in simulation 1.6416e+01
# Warning: expt average             G9:C1-C2=-8.3000e+00 is smaller than minimum value in simulation -8.2087e+00
# Warning: expt average            G12:C1-C2=-9.4000e+00 is smaller than minimum value in simulation -8.2087e+00
# Warning: expt average            G10:C3-C4=2.0700e+01 is larger than maximum value in simulation 1.6418e+01
# Warning: expt average             G9:C1-CC=1.7000e+01 is larger than maximum value in simulation 1.5801e+01
# theta: 40
# RDC scaling factors 6.5488e-01, 8.3156e-01
# RDC scaling factors 8.4150e-01, 8.8234e-01
# RDC scaling factors 1.0143e+00, 1.2204e+00
# RDC scaling factors 5.7163e-01, 9.6953e-01
# RDC scaling factors 6.1197e-03, 6.2559e-03
# Assuming weights given as minus free energies. w=exp(bias/kbt) kbt=  2.3280 
# Set non-uniform initial weights from file. Sum= 11503.331447473747 100001
# Warning: expt average         G2_H1;C3_H42=4.1957e-03 is larger than maximum value in simulation 3.7354e-03
# Warning: expt average        G2_H1';G2_H2'=6.7551e-03 is larger than maximum value in simulation 6.1478e-03
# Warning: expt average        A4_H1';C5_H1'=1.2181e-03 is larger than maximum value in simulation 3.1302e-04
# Warning: expt average        A4_H1';A4_H3'=2.1202e-03 is larger than maximum value in simulation 6.9933e-04
# Warning: expt average        A4_H61;A4_H62=6.4132e-03 is smaller than minimum value in simulation 2.1540e-02
# Warning: expt average       C5_H3';C5_HO2'=1.0834e-04 is smaller than minimum value in simulation 1.4686e-04
# Warning: expt average        U6_H4';U6_H2'=7.3339e-04 is larger than maximum value in simulation 6.9958e-04
# Warning: expt average         C8_H5;C8_H2'=7.3339e-04 is larger than maximum value in simulation 3.2350e-04
# Warning: expt average       C8_H5';C8_H5''=1.3473e-02 is smaller than minimum value in simulation 1.6214e-02
# Warning: expt average      G10_H21;G10_H22=9.5773e-03 is smaller than minimum value in simulation 2.1926e-02
# Warning: expt average       G10_H8;G10_H4'=1.5485e-03 is larger than maximum value in simulation 4.1958e-04
# Warning: expt average    G10_H5'';G10_HO2'=9.8547e-04 is larger than maximum value in simulation 5.7105e-04
# Warning: expt average       U11_H1';U11_H3=2.2999e-04 is larger than maximum value in simulation 2.1136e-04
# Warning: expt average        U11_H3;U11_H6=2.2999e-04 is larger than maximum value in simulation 1.0536e-04
# Warning: expt average       G12_H1;G12_H21=3.1190e-04 is smaller than minimum value in simulation 3.6376e-04
# Warning: expt average      G12_H1';G12_H2'=9.1405e-04 is smaller than minimum value in simulation 9.2853e-04
# Warning: expt average      C13_H1';C13_H5'=4.2992e-04 is larger than maximum value in simulation 3.0289e-04
# RDC scaling factor 6.9994e-01
# Warning: expt average              11-1H5P=0.0000e+00 is smaller than minimum value in simulation 9.9199e-01
# Warning: expt average               3-2H5P=0.0000e+00 is smaller than minimum value in simulation 9.9199e-01
# RDC scaling factor 8.5278e-01
# Warning: expt average             U7:C4p-P=1.5100e+01 is larger than maximum value in simulation 1.4951e+01
# Warning: expt average             G2:C1-C2=-1.0100e+01 is smaller than minimum value in simulation -8.3187e+00
# Warning: expt average             C3:C1-C2=-8.7000e+00 is smaller than minimum value in simulation -8.3187e+00
# Warning: expt average             A4:C1-C2=-9.0000e+00 is smaller than minimum value in simulation -8.3187e+00
# Warning: expt average             C8:C1-C2=1.7300e+01 is larger than maximum value in simulation 1.6638e+01
# Warning: expt average            G12:C1-C2=-9.4000e+00 is smaller than minimum value in simulation -8.3187e+00
# Warning: expt average            G10:C3-C4=2.0700e+01 is larger than maximum value in simulation 1.6638e+01
# Warning: expt average             G9:C1-CC=1.7000e+01 is larger than maximum value in simulation 1.6013e+01
# theta: 40
# Assuming weights given as minus free energies. w=exp(bias/kbt) kbt=  2.3280 
# Set non-uniform initial weights from file. Sum= 11503.331447473747 100001
# RDC scaling factor 1.1145e+00
# RDC scaling factor 6.1902e-01
# theta: 60
# RDC scaling factors 7.3452e-01, 7.7256e-01
# RDC scaling factors 8.5915e-01, 8.7447e-01
# RDC scaling factors 1.1145e+00, 1.3848e+00
# RDC scaling factors 6.1902e-01, 8.2643e-01
# RDC scaling factors 6.1926e-03, 6.2234e-03
# Assuming weights given as minus free energies. w=exp(bias/kbt) kbt=  2.3280 
# Set non-uniform initial weights from file. Sum= 11503.331447473747 100001
# RDC scaling factor 1.0045e+00
# RDC scaling factor 6.1697e-01
# theta: 60
# RDC scaling factors 6.9143e-01, 7.7820e-01
# RDC scaling factors 8.4708e-01, 8.9181e-01
# RDC scaling factors 1.0045e+00, 1.3694e+00
# RDC scaling factors 6.1697e-01, 1.0191e+00
# RDC scaling factors 6.1923e-03, 6.1110e-03
# Assuming weights given as minus free energies. w=exp(bias/kbt) kbt=  2.3280 
# Set non-uniform initial weights from file. Sum= 11503.331447473747 100001
# RDC scaling factor 1.0635e+00
# RDC scaling factor 6.6076e-01
# theta: 60
# RDC scaling factors 7.0720e-01, 8.2025e-01
# RDC scaling factors 8.4720e-01, 8.7867e-01
# RDC scaling factors 1.0635e+00, 1.4146e+00
# RDC scaling factors 6.6076e-01, 1.0937e+00
# RDC scaling factors 6.2253e-03, 6.1531e-03
# Assuming weights given as minus free energies. w=exp(bias/kbt) kbt=  2.3280 
# Set non-uniform initial weights from file. Sum= 11503.331447473747 100001
# RDC scaling factor 1.0143e+00
# RDC scaling factor 5.7163e-01
# theta: 60
# RDC scaling factors 6.5488e-01, 7.6599e-01
# RDC scaling factors 8.4150e-01, 8.6219e-01
# RDC scaling factors 1.0143e+00, 1.3327e+00
# RDC scaling factors 5.7163e-01, 8.7376e-01
# RDC scaling factors 6.1197e-03, 6.1888e-03
# Assuming weights given as minus free energies. w=exp(bias/kbt) kbt=  2.3280 
# Set non-uniform initial weights from file. Sum= 11503.331447473747 100001
# RDC scaling factor 1.0556e+00
# RDC scaling factor 6.1818e-01
# theta: 60
# Assuming weights given as minus free energies. w=exp(bias/kbt) kbt=  2.3280 
# Set non-uniform initial weights from file. Sum= 11503.331447473747 100001
# RDC scaling factor 6.1926e-03
# Warning: expt average              1GuaH4'=1.2939e+00 is larger than maximum value in simulation 1.2442e+00
# Warning: expt average               1GuaH8=1.7188e+00 is larger than maximum value in simulation 1.4624e+00
# Warning: expt average              3CytH41=6.9691e-02 is smaller than minimum value in simulation 8.7606e-02
# Warning: expt average              3CytH42=7.7233e-02 is smaller than minimum value in simulation 9.0288e-02
# Warning: expt average              4AdeH61=7.4677e-03 is smaller than minimum value in simulation 8.9476e-02
# Warning: expt average              4AdeH62=-4.8206e-03 is smaller than minimum value in simulation 8.5129e-02
# Warning: expt average              5CytH41=9.0368e-02 is smaller than minimum value in simulation 9.3551e-02
# Warning: expt average              5CytH42=9.1948e-02 is smaller than minimum value in simulation 1.0122e-01
# Warning: expt average               6UraH3=2.3618e+00 is larger than maximum value in simulation 1.4344e+00
# Warning: expt average              10GuaH1=6.7816e-02 is smaller than minimum value in simulation 9.2393e-02
# Warning: expt average              11UraH3=7.4565e-02 is smaller than minimum value in simulation 8.9786e-02
# Warning: expt average              12GuaH1=5.8687e-02 is smaller than minimum value in simulation 1.0239e-01
# theta: 500
# RDC scaling factors 7.3452e-01, 8.2925e-01
# RDC scaling factors 8.5915e-01, 8.6128e-01
# RDC scaling factors 1.1145e+00, 1.2631e+00
# RDC scaling factors 6.1902e-01, 9.4488e-01
# RDC scaling factors 6.1926e-03, 6.4906e-03
# Assuming weights given as minus free energies. w=exp(bias/kbt) kbt=  2.3280 
# Set non-uniform initial weights from file. Sum= 11503.331447473747 100001
# RDC scaling factor 6.1923e-03
# Warning: expt average              1GuaH4'=1.2939e+00 is larger than maximum value in simulation 1.2491e+00
# Warning: expt average               1GuaH8=1.7188e+00 is larger than maximum value in simulation 1.3941e+00
# Warning: expt average              3CytH41=6.9691e-02 is smaller than minimum value in simulation 8.7052e-02
# Warning: expt average              3CytH42=7.7233e-02 is smaller than minimum value in simulation 8.6624e-02
# Warning: expt average              4AdeH61=7.4677e-03 is smaller than minimum value in simulation 8.5442e-02
# Warning: expt average              4AdeH62=-4.8206e-03 is smaller than minimum value in simulation 8.4674e-02
# Warning: expt average              5CytH41=9.0368e-02 is smaller than minimum value in simulation 9.4198e-02
# Warning: expt average              5CytH42=9.1948e-02 is smaller than minimum value in simulation 9.7579e-02
# Warning: expt average               6UraH3=2.3618e+00 is larger than maximum value in simulation 1.4859e+00
# Warning: expt average              10GuaH1=6.7816e-02 is smaller than minimum value in simulation 9.5436e-02
# Warning: expt average              11UraH3=7.4565e-02 is smaller than minimum value in simulation 9.3114e-02
# Warning: expt average              12GuaH1=5.8687e-02 is smaller than minimum value in simulation 9.0872e-02
# theta: 500
# RDC scaling factors 6.9143e-01, 7.6729e-01
# RDC scaling factors 8.4708e-01, 8.6471e-01
# RDC scaling factors 1.0045e+00, 1.1393e+00
# RDC scaling factors 6.1697e-01, 7.0681e-01
# RDC scaling factors 6.1923e-03, 6.4513e-03
# Assuming weights given as minus free energies. w=exp(bias/kbt) kbt=  2.3280 
# Set non-uniform initial weights from file. Sum= 11503.331447473747 100001
# RDC scaling factor 6.2253e-03
# Warning: expt average              1GuaH4'=1.2939e+00 is larger than maximum value in simulation 1.2473e+00
# Warning: expt average               1GuaH8=1.7188e+00 is larger than maximum value in simulation 1.4919e+00
# Warning: expt average              3CytH41=6.9691e-02 is smaller than minimum value in simulation 9.2582e-02
# Warning: expt average              3CytH42=7.7233e-02 is smaller than minimum value in simulation 8.9899e-02
# Warning: expt average              4AdeH61=7.4677e-03 is smaller than minimum value in simulation 8.9463e-02
# Warning: expt average              4AdeH62=-4.8206e-03 is smaller than minimum value in simulation 8.5137e-02
# Warning: expt average              5CytH41=9.0368e-02 is smaller than minimum value in simulation 9.0995e-02
# Warning: expt average              6UraH1'=1.2072e-01 is smaller than minimum value in simulation 1.2081e-01
# Warning: expt average               6UraH3=2.3618e+00 is larger than maximum value in simulation 1.4276e+00
# Warning: expt average              10GuaH1=6.7816e-02 is smaller than minimum value in simulation 9.5340e-02
# Warning: expt average             10GuaH21=1.0203e-01 is smaller than minimum value in simulation 1.0803e-01
# Warning: expt average              11UraH3=7.4565e-02 is smaller than minimum value in simulation 8.8729e-02
# Warning: expt average              12GuaH1=5.8687e-02 is smaller than minimum value in simulation 1.0089e-01
# theta: 500
# RDC scaling factors 7.0720e-01, 7.6142e-01
# RDC scaling factors 8.4720e-01, 8.5796e-01
# RDC scaling factors 1.0635e+00, 1.2819e+00
# RDC scaling factors 6.6076e-01, 7.5579e-01
# RDC scaling factors 6.2253e-03, 6.4647e-03
# Assuming weights given as minus free energies. w=exp(bias/kbt) kbt=  2.3280 
# Set non-uniform initial weights from file. Sum= 11503.331447473747 100001
# RDC scaling factor 6.1197e-03
# Warning: expt average              1GuaH4'=1.2939e+00 is larger than maximum value in simulation 1.2052e+00
# Warning: expt average               1GuaH8=1.7188e+00 is larger than maximum value in simulation 1.4741e+00
# Warning: expt average              3CytH41=6.9691e-02 is smaller than minimum value in simulation 8.4812e-02
# Warning: expt average              3CytH42=7.7233e-02 is smaller than minimum value in simulation 8.6887e-02
# Warning: expt average              4AdeH61=7.4677e-03 is smaller than minimum value in simulation 8.0663e-02
# Warning: expt average              4AdeH62=-4.8206e-03 is smaller than minimum value in simulation 7.7805e-02
# Warning: expt average              6UraH1'=1.2072e-01 is smaller than minimum value in simulation 1.2232e-01
# Warning: expt average             6Ura1H2'=1.5312e-01 is smaller than minimum value in simulation 1.6002e-01
# Warning: expt average               6UraH3=2.3618e+00 is larger than maximum value in simulation 1.4207e+00
# Warning: expt average              10GuaH1=6.7816e-02 is smaller than minimum value in simulation 8.9212e-02
# Warning: expt average             10GuaH21=1.0203e-01 is smaller than minimum value in simulation 1.0318e-01
# Warning: expt average              11UraH3=7.4565e-02 is smaller than minimum value in simulation 8.3766e-02
# Warning: expt average              12GuaH1=5.8687e-02 is smaller than minimum value in simulation 9.5779e-02
# theta: 500
# RDC scaling factors 6.5488e-01, 7.7448e-01
# RDC scaling factors 8.4150e-01, 8.4837e-01
# RDC scaling factors 1.0143e+00, 1.3329e+00
# RDC scaling factors 5.7163e-01, 7.9448e-01
# RDC scaling factors 6.1197e-03, 6.5090e-03
# Assuming weights given as minus free energies. w=exp(bias/kbt) kbt=  2.3280 
# Set non-uniform initial weights from file. Sum= 11503.331447473747 100001
# RDC scaling factor 6.1901e-03
# Warning: expt average              1GuaH4'=1.2939e+00 is larger than maximum value in simulation 1.2487e+00
# Warning: expt average               1GuaH8=1.7188e+00 is larger than maximum value in simulation 1.4910e+00
# Warning: expt average              3CytH41=6.9691e-02 is smaller than minimum value in simulation 8.5788e-02
# Warning: expt average              3CytH42=7.7233e-02 is smaller than minimum value in simulation 8.6593e-02
# Warning: expt average              4AdeH61=7.4677e-03 is smaller than minimum value in simulation 8.1591e-02
# Warning: expt average              4AdeH62=-4.8206e-03 is smaller than minimum value in simulation 7.8700e-02
# Warning: expt average               6UraH3=2.3618e+00 is larger than maximum value in simulation 1.4853e+00
# Warning: expt average              10GuaH1=6.7816e-02 is smaller than minimum value in simulation 9.0239e-02
# Warning: expt average              11UraH3=7.4565e-02 is smaller than minimum value in simulation 8.4730e-02
# Warning: expt average              12GuaH1=5.8687e-02 is smaller than minimum value in simulation 9.0839e-02
# theta: 500
```

# appendix: calculating the final bias potential for each frame¶

A first step we take the average HILLS file

`bash ../script/medie.sh < data/HILLS.0 200000 1000000 > data/HILLS.0.avg`

Then, calculate the bias for each frame using the following `plumed.weights.dat` file.

```
RESTART
MOLINFO STRUCTURE=uucg_gmx.pdb
WHOLEMOLECULES ENTITY0=1-447
ee3: ERMSD ATOMS=@lcs-1,@lcs-2,@lcs-3,@lcs-4,@lcs-5,@lcs-6,@lcs-7,@lcs-8,@lcs-9,@lcs-10,@lcs-11,@lcs-12,@lcs-13,@lcs-14 REFERENCE=2koc_gmx.pdb CUTOFF=3.2
metad: METAD ARG=ee3 PACE=500000001 HEIGHT=0.0 SIGMA=0.1 FILE=HILLS.0.avg TEMP=280 BIASFACTOR=15 GRID_MIN=0.0 GRID_MAX=6.0
bias: REWEIGHT_BIAS TEMP=280
PRINT ARG=metad.bias FILE=bias STRIDE=1 RESTART=NO
```

And run plumed driver

`plumed driver --plumed plumed.weights.dat --mf_xtc traj_temp_f_0.xtc`

This produces a file `bias` that contains the averaged final bias, and the weights can be estimated as $w\_i \propto exp(bias/kt)$
