# Supplementary material for: Integrating NMR and simulations reveals motions in the UUCG tetraloop
Source: Nucleic Acids Res. 2020 May 19;48(11):5839–48. doi: 10.1093/nar/gkaa399 (PMC7293013; doi:10.1093/nar/gkaa399)
Supplement: gkaa399_Supplemental_Files [file gkaa399_supplemental_files.zip › SI4_random_forest.html]

SI4\_random\_forest


In this notebook we investigate the differences between the native state (state A, eRMSD from native less than 0.7) compared to state B. In our MD+eNOE ensemble, state B consists in non-native loop conformations.

In this analysis we train a random forest classifier in the space of different observables (angles, distances, etc) that can discriminate state A from state B. Training and test examples are bootstraped from the optimal MD+set A distribution.

The classifier returns a score for each input feature. The higher the score, the more important the corresponding feature for classification.

In [1]:

```
# import libraries
import barnaba as bb
from barnaba import definitions
import numpy as np
import pickle
import os
import matplotlib.pyplot as plt
np.random.seed(144)
```

In [2]:

```
# read weights from file
theta=20
data_tmp = np.array([[float(x) for x in line.split()] for line in open("data/my_theta/weights_set_A_t_%d.dat" % (theta),"r")])
weights = data_tmp[:,1]

# draw 50k samples 
nsamples = 50000
ff = np.random.choice(len(weights),size=50000,p=weights/np.sum(weights),replace=True)

# split between state  A and B
ermsd_full = pickle.load(open("data/observables/ermsd_full.p",'rb'), encoding='bytes')
ermsd_new = ermsd_full[ff]
ii = np.where(ermsd_new<0.7)[0]
jj = np.where(ermsd_new>=0.7)[0]
bb= len(ii)+len(jj)
```

# define the random forest classifier, using sklearn implementation¶

In [3]:

```
from sklearn.ensemble import RandomForestClassifier
import pandas as pd
import numpy as np
from sklearn import metrics


def rfc(x,y,labels,outfile,train_fraction=0.8,max_depth=2):

    x = np.array(x)
    y = np.array(y)
    # do some sanity checks
    assert(y.shape[0]==x.shape[0])
    assert(len(labels)==x.shape[1])

    # shuffle and split in training/test set
    randomize = np.arange(len(y))
    np.random.shuffle(randomize)
    stop_train = int(train_fraction*(y.shape[0]))

    x_train = x[randomize][:stop_train,:]
    y_train = y[randomize][:stop_train]

    x_test = x[randomize][stop_train:,:]
    y_test = y[randomize][stop_train:]
    print("# number of training examples: %d" % x_train.shape[0])
    print("# number of test     examples: %d" % x_test.shape[0])
    print("# number of input features:    %d" % x_test.shape[1])


    # define classifier
    clf = RandomForestClassifier(n_estimators=100,n_jobs=20,max_depth=max_depth,random_state=2)
    
    clf.fit(x_train,y_train)
    y_pred_train=clf.predict(x_train)
    y_pred_test=clf.predict(x_test)
    str1 = "# Training accuracy %5.3f (%s)\n" % (metrics.accuracy_score(y_train,y_pred_train),x_train.shape)
    str1 += "# Test     accuracy %5.3f (%s)" % (metrics.accuracy_score(y_test,y_pred_test),x_test.shape)
    str2 = "# Training F1 score %5.3f (%s)\n" % (metrics.f1_score(y_train,y_pred_train),x_train.shape)
    str2 += "# Test     F1 score %5.3f (%s)" % (metrics.f1_score(y_test,y_pred_test),x_test.shape)
    print(str1)
    print(str2)
    #fh = open(outfile,"w")
    #fh.write(str1 + "\n")
    #fh.write(str2 + "\n")
    sorted_imp = np.argsort(clf.feature_importances_)[::-1]

    my_idx = []
    yy = clf.feature_importances_/np.max(clf.feature_importances_)
    for i,el in enumerate(sorted_imp):
        str3 = "%4d %4d %20s %6.4f " % (i,el,labels[el],yy[el])
        if(i<21): print(str3)
        if(yy[el]>0.2): my_idx.append(el)
    #    fh.write(str3+"\n")
    #fh.close()
    #stats = [metrics.accuracy_score(y_train,y_pred_train),metrics.f1_score(y_train,y_pred_train),\
    #        metrics.accuracy_score(y_test,y_pred_test),metrics.f1_score(y_test,y_pred_test)]
    stats = []

    return stats, my_idx
```

# train a Random Forest with torsion angles as input features¶

In [4]:

```
# read pickle from file
angles,res = pickle.load(open("data/observables/angles.p",'rb'), encoding='bytes')
mask = np.ones(14*7, dtype=bool)
# mask nans at the termini
mask[0]= False
mask[1] = False
mask[-2] = False
mask[-3] = False
# reshape
ll = angles.shape[0]
angles = angles.reshape(ll,14*7)[:,mask]

# bootstrap using the indeces crated above
angles_new = angles[ff,:]
# split in state A and B
angles_A = angles_new[ii,:]
angles_B = angles_new[jj,:]
```

In [5]:

```
# define labels to make it human-readable
labels = [["%s_%s" % (i,el) for el in definitions.bb_angles] for i in res]
l = 0
labels1 = []
for j in range(len(labels)):
    for k in range(len(labels[j])):
        if(mask[l]): labels1.append(labels[j][k])
        l+=1

X = np.vstack([angles_A,angles_B])
Y = np.hstack([np.ones(len(angles_A)), np.zeros(len(angles_B))])
print(X.shape,Y.shape,len(labels1))
stats = rfc(X,Y,labels=labels1,outfile="crap",max_depth=3)
```

```
(50000, 94) (50000,) 94
# number of training examples: 40000
# number of test     examples: 10000
# number of input features:    94
# Training accuracy 0.989 ((40000, 94))
# Test     accuracy 0.988 ((10000, 94))
# Training F1 score 0.994 ((40000, 94))
# Test     F1 score 0.993 ((10000, 94))
   0   52           C_8_0_zeta 1.0000 
   1   59           G_9_0_zeta 0.6545 
   2   51            C_8_0_eps 0.5142 
   3   50          C_8_0_delta 0.3281 
   4   43          U_7_0_delta 0.2712 
   5   44            U_7_0_eps 0.2371 
   6   38           U_6_0_zeta 0.2321 
   7   45           U_7_0_zeta 0.2041 
   8   54          G_9_0_alpha 0.1885 
   9   62          G_10_0_beta 0.1823 
  10   40          U_7_0_alpha 0.1568 
  11   60            G_9_0_chi 0.1024 
  12   47          C_8_0_alpha 0.0904 
  13   49          C_8_0_gamma 0.0731 
  14   53            C_8_0_chi 0.0706 
  15   57          G_9_0_delta 0.0698 
  16   63         G_10_0_gamma 0.0579 
  17   56          G_9_0_gamma 0.0375 
  18   42          U_7_0_gamma 0.0293 
  19   61         G_10_0_alpha 0.0197 
  20   68         U_11_0_alpha 0.0133
```

We can see that C8 zeta and C9 zeta are the most important features for classification. We plot below the corresponding histograms

In [6]:

```
# read data for 2koc and 6by5 as a reference
angles_2koc,res = pickle.load(open("data/observables/angles_2koc.p",'rb'), encoding='bytes')
angles_6by5,res = pickle.load(open("data/observables/angles_6by5.p",'rb'), encoding='bytes')

# initialize plot
fig,ax = plt.subplots(2,1,figsize=(3.5,5),sharex=True)
plt.subplots_adjust(left=0.15, bottom=0.12, right=0.95, top=0.96, wspace=0.25, hspace=0.1)

bins = np.linspace(-np.pi, np.pi,50)
z = angles_A.shape[0]+angles_B.shape[0]

hh,ee = np.histogram(angles_A[:,52],bins=bins)
xx = (ee[1:]+ee[:-1])*0.5
hh1,ee1 = np.histogram(angles_B[:,52],bins=bins)
xx1 = (ee1[1:]+ee1[:-1])*0.5

ax[0].plot(xx,hh/z,c='#2c6fbb',ls="-",lw=1.5)
ax[0].fill_between(xx1,hh1/z,color='#2c6fbb',ls="-",lw=1.5,alpha=0.7)
ax[0].scatter(angles_2koc[:,7,5],[-0.01]*len(angles_2koc),c='red')
ax[0].scatter(angles_6by5[:,7,5],[-0.01]*len(angles_6by5),c='orange')
ax[0].text(-2.5,0.22,"C8, $\zeta$",fontsize=12)
hh,ee = np.histogram(angles_A[:,59],bins=bins)
xx = (ee[1:]+ee[:-1])*0.5
hh1,ee1 = np.histogram(angles_B[:,59],bins=bins)
xx1 = (ee1[1:]+ee1[:-1])*0.5

ax[1].plot(xx,hh/z,c='#2c6fbb',ls="-",lw=2)
ax[1].fill_between(xx1,hh1/z,color='#2c6fbb',ls="-",lw=2,alpha=0.7)
ax[1].set_xticks([-np.pi,-np.pi/2,0,np.pi/2.,np.pi])
oo = ax[1].set_xticklabels(["$-\pi$","$-\pi/2$","0","$\pi/2$","$\pi$"])
ax[1].scatter(angles_2koc[:,8,5],[-0.01]*len(angles_2koc),c='#be0119',marker="o")
ax[1].scatter(angles_6by5[:,8,5],[-0.01]*len(angles_6by5),c='orange',marker="x")
ax[1].text(2,0.12,"G9, $\zeta$",fontsize=12)
ax[1].set_xlabel("Torsion angle")
ax[0].set_yticks([0,0.1,0.2])
#plt.savefig("figures/angles_hist.pdf")
#plt.savefig("figures/angles_hist.png",dpi=600)
#plt.close()
```

Out[6]:

```
[<matplotlib.axis.YTick at 0x7f42fc495d30>,
 <matplotlib.axis.YTick at 0x7f42fc495668>,
 <matplotlib.axis.YTick at 0x7f4308c97a58>]
```

# train a Random Forest with distances between center of 6-memeberd rings as input features¶

In [7]:

```
# read distance vectors from pickle
rvecs,res = pickle.load(open("data/observables/rvecs.p",'rb'), encoding='bytes')

ll = rvecs.shape[0]

ii_t = np.triu_indices(14,1)
rdist1 = np.sqrt((rvecs**2).sum(axis=3))
ids = np.arange(ll)
# take entries from upper diagonal
rdist = np.array([rdist1[el][ii_t] for el in ids])

# bootstrap
rdist_new = rdist[ff]
rvecs_A = rdist_new[ii,:]
rvecs_B = rdist_new[jj,:]
```

We do not use the distances directly, but a switching function of the distance

In [8]:

```
def switch(xx,a=0.6):
    r6 = (xx/a)**6
    r12 = r6*r6
    return (1.-r6)/(1.-r12)

labels_rvecs = []
for i,el in enumerate(res):
    for j in range(i+1,len(res)):
        labels_rvecs.append("%s:%s" % (el,res[j]))
X = np.vstack([rvecs_A,rvecs_B])
X = np.vstack([switch(rvecs_A),switch(rvecs_B)])

Y = np.hstack([np.ones(len(rvecs_A)), np.zeros(len(rvecs_B))])
print(X.shape,Y.shape,len(labels_rvecs))
stats = rfc(X,Y,labels=labels_rvecs,outfile="crap",max_depth=3)
```

```
(50000, 91) (50000,) 91
# number of training examples: 40000
# number of test     examples: 10000
# number of input features:    91
# Training accuracy 0.999 ((40000, 91))
# Test     accuracy 0.999 ((10000, 91))
# Training F1 score 0.999 ((40000, 91))
# Test     F1 score 0.999 ((10000, 91))
   0   56          U_6_0:C_8_0 1.0000 
   1   76         G_9_0:G_10_0 0.8202 
   2   57          U_6_0:G_9_0 0.7016 
   3   49          C_5_0:G_9_0 0.6224 
   4   77         G_9_0:U_11_0 0.5425 
   5   70          C_8_0:G_9_0 0.4669 
   6   39          A_4_0:C_8_0 0.4035 
   7   64          U_7_0:G_9_0 0.3573 
   8   78         G_9_0:G_12_0 0.3358 
   9   40          A_4_0:G_9_0 0.2446 
  10   30          C_3_0:G_9_0 0.2392 
  11   48          C_5_0:C_8_0 0.1755 
  12   79         G_9_0:C_13_0 0.1361 
  13   71         C_8_0:G_10_0 0.1188 
  14   29          C_3_0:C_8_0 0.0930 
  15   18          G_2_0:C_8_0 0.0548 
  16   80         G_9_0:C_14_0 0.0516 
  17   63          U_7_0:C_8_0 0.0440 
  18   73         C_8_0:G_12_0 0.0362 
  19   19          G_2_0:G_9_0 0.0359 
  20   67         U_7_0:G_12_0 0.0327
```

U6-C8,G9-G10 and U6-G9 are the most important features. We then make a 2-d histogram that show the distribution of U6-C8 and U6-G9, the latter one reporting directly on the U6-G9 interaction.

In [9]:

```
from scipy import stats


datax = np.sqrt(np.sum(rvecs[:,5,7,:]**2,axis=1))
datay = np.sqrt(np.sum(rvecs[:,5,8,:]**2,axis=1))
xa=np.arange(1.05*np.min(datax),1.05*np.max(datax),0.025)
ya=np.arange(1.05*np.min(datay),1.05*np.max(datay),0.025)
#print(datax.shape,(weights_0.reshape(-1)).shape)
kernel = stats.gaussian_kde(np.array([datax,datay]),weights=weights,bw_method=0.15)

X, Y = np.meshgrid(xa,ya)
grid_coords = np.vstack([X.ravel(), Y.ravel()])
H = np.reshape(kernel(grid_coords).T, X.shape)
```

In [10]:

```
# vectors connecting center of nucleobases
fname = "data/observables/rvecs_2koc.p"
[rvecs_2koc,res_2koc] = pickle.load(open(fname,'rb'), encoding='bytes')

fname = "data/observables/rvecs_6by5.p"
[rvecs_6by5,res_6by5] = pickle.load(open(fname,'rb'), encoding='bytes')

d_69_2koc = np.sqrt(np.sum(rvecs_2koc[:,5,8,:]**2,axis=1))
d_69_6by5 = np.sqrt(np.sum(rvecs_6by5[:,5,8,:]**2,axis=1))

d_68_2koc = np.sqrt(np.sum(rvecs_2koc[:,5,7,:]**2,axis=1))
d_68_6by5 = np.sqrt(np.sum(rvecs_6by5[:,5,7,:]**2,axis=1))

Z= -(np.log(H))
Z -= np.min(Z)

import matplotlib.pyplot as plt
import matplotlib.patches as patches

import seaborn as sns 

levels = [0,1,2,3,4,5,6,7,8]
sns.set_palette(sns.color_palette("cubehelix", len(levels)-1))
cmap = sns.cubehelix_palette(len(levels)-1, as_cmap=True,reverse=True,rot=-0.2)

fig,ax = plt.subplots(1,1,figsize=(5., 5.5))


plt.subplots_adjust(left=0.14, bottom=0.1, right=0.98, top=0.98, wspace=0.07, hspace=0.1)

plt.xlim(0.3,2.)
plt.ylim(0.3,2.2)
CS = ax.contourf(X, Y, Z,levels=levels,cmap=cmap)
CS1 = ax.contour(X, Y, Z,levels=levels,colors='k',linewidths=0.3)

CB = fig.colorbar(CS,orientation="horizontal")

plt.scatter(d_68_2koc,d_69_2koc,s=20,c='#be0119',label='2KOC',marker='o',zorder=10)
plt.scatter(d_68_6by5,d_69_6by5,s=60,c='orange',label='6BY5',marker="x",zorder=10)
plt.legend(ncol=1,loc="lower right")
#plt.xlabel("eRMSD from native (residues 4-11)")
plt.ylabel("U6-G9 distance [nm]")
plt.xlabel("U6-C8 distance [nm]")
plt.xticks([0.5,1,1.5,2])
plt.yticks([0.5,1,1.5,2])
#plt.savefig("figures/2d_projection.png",dpi=600)
#plt.savefig("figures/2d_projection.pdf")
plt.show()
#plt.close()
#plt.figure(figsize=(8,8))
#plt.scatter(rvecs_A[:,56], rvecs_A[:,57],s=1.5,zorder=10)
#lt.scatter(rvecs_B[:,56], rvecs_B[:,57],s=1.5,zorder=10)
```

# Train a Random Forest with experimental data as input features¶

Here we perform an analysis identical to the one performed before. The difference is that we use as input features the experimental data. This allows us to determine the experimental observations that are most sensitive to the presence of state B. We inspect them in detail and compare them with the reference ensemble 2KOC.

In [11]:

```
import pandas as pd
from scipy import stats

# define a function that returns the labels fo the most important fatures ( more than 0.2)
def analyse(dirname,name):
    data_calc = pd.read_csv("data/calc/%s/%s.calc.dat" % (dirname,name),header=None,sep="\s+",index_col=0,comment="#")

    data_pdb = pd.read_csv("data/calc/%s/2koc.%s.calc.dat" % (dirname,name),header=None,sep="\s+",index_col=0,comment="#")
    data_exp = pd.read_csv("data/exp/%s/%s.exp.dat" % (dirname,name),names=["label","exp","sigma"],sep="\s+",comment="#")
    labels = data_exp["label"].values

    if("NOE" in name):
        data_new = np.power(data_calc.values[ff,:],-1/6.)
        data_pdb = np.power(data_pdb.values,-1/6.)
    else:
        data_new = data_calc.values[ff,:]
        data_pdb = data_pdb.values
        
    # bootstrap
    data_B = data_new[jj,:]
    data_A = data_new[ii,:]
    X = np.vstack([data_A,data_B])
    Y = np.hstack([np.ones(len(data_A)), np.zeros(len(data_B))])
    data = []
    
    # train the classifier
    stats,sorted_idx = rfc(X,Y,labels=labels,outfile="%s/%s.rf" % (dirname,name),max_depth=2)
    labels_sorted = []
    for j in sorted_idx:
        full = data_new[:,j].mean()
        sA = data_A[:,j].mean()
        sB = data_B[:,j].mean()
        v2koc = data_pdb[:,j].mean()
        if("NOE" in name):
            full = np.power(full,-6)
            sA = np.power(sA,-6)
            sB = np.power(sB,-6)
            v2koc = np.power(v2koc,-6)
        stri = "%20s %4.2f %4.2f %4.2f %4.2f %4.2f" % (labels[j],data_exp.values[j,1],full,sA,sB,v2koc)
        print(stri)
        data.append([data_exp.values[j,1],data_exp.values[j,2],full,sA,sB,v2koc])
        labels_sorted.append(labels[j])
    return np.array(data), labels_sorted
```

In [12]:

```
# here we plot the calculated/experimental values, together with  the value from 2KOC
files = [["set_A/","eNOE_unidir"],["set_A/","eNOE"],\
         ["set_B/","NOE"],["set_B/","J3"],\
         ["set_B/","CCRR"],["set_B/","RDC"],\
         ["set_C/","RDC1"],["set_C/","RDC2"],["set_D/","sPRE"]]


rwn = "set_A"
theta=20
suspects = []
for el in files:
    data, labels = analyse(el[0],el[1])
    print(labels)
    calc = np.ones((len(labels),4))*1e10
    for k in range(4):
        nmf = "data/my_theta/%s/rw_%s_%s_theta_%d_rep_%d.stats.dat"  % (rwn,el[0].split("/")[-2],el[1],theta,k)
        df = pd.read_csv(nmf,header=None,sep="\s+",index_col=0,comment="#")
        for il,lab in enumerate(labels):
            calc[il,k] = df.loc[lab].values[-1]
    data_pdbf = "data/my_theta/%s/2koc_%s.stats.dat"  % (el[0].split("/")[-2],el[1])
    #data_pdbf = "data/comparison/%s/6by5_%s.stats.dat"  % (el[0].split("/")[-2],el[1])
    
    df = pd.read_csv(data_pdbf,header=None,sep="\s+",index_col=0,comment="#")
    data_pdb = np.ones(len(labels))*1e10
    for il,lab in enumerate(labels):
        data_pdb[il] = df.loc[lab].values[-1]
    xx = np.arange(data.shape[0])
    plt.errorbar(xx,data[:,0],yerr=data[:,1],fmt="o",label="exp",c="r") 
    plt.errorbar(xx,calc.mean(axis=1),yerr=calc.std(axis=1,ddof=1),fmt="s",label="calc")

    plt.scatter(xx,data_pdb,label="2KOC",c="y")
    for pp in range(len(labels)):
        #diff1 = np.abs(data[pp,0]-calc.mean(axis=1)[pp])/data[pp,1]
        #diff2 = np.abs(data[pp,0]-data_pdb[pp])/data[pp,1]
        #if(diff1>0.0 or diff2>0.0):
        suspects.append(["%s-%s" % (el[1],labels[pp]), data[pp,0], data[pp,1],calc.mean(axis=1)[pp],data_pdb[pp],calc.std(axis=1,ddof=1)[pp]])
        print(suspects[-1])
    plt.xticks(xx,labels,rotation=75)
    #plt.legend()
    plt.show()
    plt.close()
```

```
# number of training examples: 40000
# number of test     examples: 10000
# number of input features:    177
# Training accuracy 0.999 ((40000, 177))
# Test     accuracy 0.999 ((10000, 177))
# Training F1 score 0.999 ((40000, 177))
# Test     F1 score 0.999 ((10000, 177))
   0   12          C8_H6;G9_H1 1.0000 
   1   83         U6_H1';G9_H1 0.9781 
   2  157        U6_H3';C8_H41 0.8627 
   3   38         U6_H2';C8_H5 0.8135 
   4   10         C8_H1';G9_H1 0.7080 
   5    7         G9_H1;G10_H8 0.6567 
   6    2          U6_H6;G9_H1 0.6511 
   7   45        C8_H4';G9_H3' 0.6244 
   8   11          C8_H5;G9_H1 0.5840 
   9    5         G9_H1;G10_H1 0.5817 
  10   19         C8_H5";G9_H1 0.5356 
  11   16         U6_H6;C8_H41 0.4990 
  12  154       C14_H41;C14_H5 0.4830 
  13  117         U6_H2';C8_H6 0.3266 
  14   48       G9_H2';G10_H4' 0.2602 
  15  163        C8_H4';G9_H5' 0.2599 
  16  155       C14_H42;C14_H6 0.2220 
  17   77       G10_H5";G10_H8 0.2219 
  18   88         U7_H2';G9_H1 0.2109 
  19  150        U7_H5";C8_H41 0.1628 
  20    6        G9_H1;G10_H1' 0.1302 
         C8_H6;G9_H1 4.38 4.72 4.06 11.86 4.25
        U6_H1';G9_H1 3.43 4.15 3.41 14.38 3.46
       U6_H3';C8_H41 3.61 4.61 4.02 10.68 3.52
        U6_H2';C8_H5 2.72 2.85 2.47 6.90 2.72
        C8_H1';G9_H1 4.15 4.52 3.94 10.47 4.04
        G9_H1;G10_H8 5.08 6.32 5.84 10.07 5.73
         U6_H6;G9_H1 5.53 7.01 6.07 16.75 5.99
       C8_H4';G9_H3' 3.77 4.62 4.33 6.69 3.83
         C8_H5;G9_H1 5.26 5.55 4.81 13.32 5.00
        G9_H1;G10_H1 4.96 5.71 4.92 14.16 5.09
        C8_H5";G9_H1 5.63 7.16 6.67 10.86 6.44
        U6_H6;C8_H41 3.42 4.92 4.29 11.46 3.88
      C14_H41;C14_H5 3.59 3.46 3.62 2.70 3.69
        U6_H2';C8_H6 3.80 4.18 3.86 6.73 3.95
      G9_H2';G10_H4' 2.97 3.00 2.65 6.37 3.00
       C8_H4';G9_H5' 3.21 4.08 3.92 5.08 4.04
      C14_H42;C14_H6 4.37 4.69 4.62 5.11 4.64
      G10_H5";G10_H8 2.95 2.84 2.69 3.95 3.06
        U7_H2';G9_H1 4.41 6.06 5.28 14.04 4.80
['C8_H6;G9_H1', "U6_H1';G9_H1", "U6_H3';C8_H41", "U6_H2';C8_H5", "C8_H1';G9_H1", 'G9_H1;G10_H8', 'U6_H6;G9_H1', "C8_H4';G9_H3'", 'C8_H5;G9_H1', 'G9_H1;G10_H1', 'C8_H5";G9_H1', 'U6_H6;C8_H41', 'C14_H41;C14_H5', "U6_H2';C8_H6", "G9_H2';G10_H4'", "C8_H4';G9_H5'", 'C14_H42;C14_H6', 'G10_H5";G10_H8', "U7_H2';G9_H1"]
['eNOE_unidir-C8_H6;G9_H1', 4.38, 0.657, 4.018000000000001, 4.225, 0.041448763552125435]
["eNOE_unidir-U6_H1';G9_H1", 3.43, 0.5145, 3.38975, 3.4389999999999996, 0.16839314119048926]
["eNOE_unidir-U6_H3';C8_H41", 3.61, 0.5415, 3.769, 3.51, 0.19424211695716273]
["eNOE_unidir-U6_H2';C8_H5", 2.72, 0.408, 2.45325, 2.69, 0.0516034559566184]
["eNOE_unidir-C8_H1';G9_H1", 4.15, 0.6225, 3.98625, 4.002, 0.15622713165985805]
['eNOE_unidir-G9_H1;G10_H8', 5.08, 0.762, 5.8375, 5.718999999999999, 0.2182681836640422]
['eNOE_unidir-U6_H6;G9_H1', 5.53, 0.8295, 6.24525, 5.985, 0.21581994810489624]
["eNOE_unidir-C8_H4';G9_H3'", 3.77, 0.5655, 4.31875, 3.821, 0.1445830211331884]
['eNOE_unidir-C8_H5;G9_H1', 5.26, 0.789, 4.786249999999999, 4.978, 0.10434677762154407]
['eNOE_unidir-G9_H1;G10_H1', 4.96, 0.7440000000000001, 4.954, 5.073, 0.17439610087384388]
['eNOE_unidir-C8_H5";G9_H1', 5.63, 0.8445, 6.4182500000000005, 6.438, 0.23505371726479862]
['eNOE_unidir-U6_H6;C8_H41', 3.42, 0.513, 4.19975, 3.8310000000000004, 0.1704980449545781]
['eNOE_unidir-C14_H41;C14_H5', 3.59, 0.5385, 3.1192499999999996, 3.6860000000000004, 0.3432184678403344]
["eNOE_unidir-U6_H2';C8_H6", 3.8, 0.57, 3.80975, 3.945, 0.08020962120178181]
["eNOE_unidir-G9_H2';G10_H4'", 2.97, 0.4455, 2.63775, 2.977, 0.13578751783577186]
["eNOE_unidir-C8_H4';G9_H5'", 3.21, 0.4815, 3.83725, 4.026, 0.0967793883014353]
['eNOE_unidir-C14_H42;C14_H6', 4.37, 0.6555, 4.7135, 4.638999999999999, 0.1594605489350476]
['eNOE_unidir-G10_H5";G10_H8', 2.95, 0.4425, 2.5685000000000002, 3.056, 0.060274372663678626]
["eNOE_unidir-U7_H2';G9_H1", 4.41, 0.6615, 5.3115000000000006, 4.788, 0.17770856291505352]
```

```
# number of training examples: 40000
# number of test     examples: 10000
# number of input features:    63
# Training accuracy 0.997 ((40000, 63))
# Test     accuracy 0.997 ((10000, 63))
# Training F1 score 0.998 ((40000, 63))
# Test     F1 score 0.998 ((10000, 63))
   0   11        U6_HO2';G9_H1 1.0000 
   1   59         C8_H5';G9_H8 0.7071 
   2   12         U6_H2';G9_H1 0.6566 
   3   30        U6_H2';C8_H41 0.4968 
   4   39         C8_H4';G9_H8 0.3939 
   5   58         C8_H5";G9_H8 0.3880 
   6    0        C8_H4';G10_H8 0.3306 
   7   32       C13_H42;C13_H5 0.2232 
   8   21       C13_H41;C13_H5 0.1363 
   9   56        C8_H4';C8_H5" 0.1287 
  10   14        U6_HO2';U7_H6 0.1231 
  11   25        G2_H1;C13_H42 0.1139 
  12   24        G2_H1;C13_H41 0.0791 
  13   13       U6_H2';U6_HO2' 0.0639 
  14   36         U6_H2';U7_H6 0.0501 
  15   41        G10_H8;U11_H5 0.0265 
  16   43          C5_H6;U6_H6 0.0202 
  17    7         U6_H6;G10_H1 0.0195 
  18   22         C5_H41;C5_H5 0.0172 
  19   31         C5_H42;C5_H5 0.0130 
  20   37         U6_H2';U6_H6 0.0071 
       U6_HO2';G9_H1 3.13 4.10 3.37 14.31 3.08
        C8_H5';G9_H8 4.86 4.83 4.29 9.76 3.67
        U6_H2';G9_H1 3.55 4.60 3.84 14.32 3.54
       U6_H2';C8_H41 3.73 4.26 3.75 9.24 3.65
        C8_H4';G9_H8 5.48 5.36 4.94 8.74 4.85
        C8_H5";G9_H8 5.17 5.75 5.33 8.94 4.86
       C8_H4';G10_H8 5.77 7.27 7.49 6.21 6.90
      C13_H42;C13_H5 2.62 2.47 2.43 2.72 2.43
["U6_HO2';G9_H1", "C8_H5';G9_H8", "U6_H2';G9_H1", "U6_H2';C8_H41", "C8_H4';G9_H8", 'C8_H5";G9_H8', "C8_H4';G10_H8", 'C13_H42;C13_H5']
["eNOE-U6_HO2';G9_H1", 3.13, 0.313, 3.371, 3.075, 0.10611314715905851]
["eNOE-C8_H5';G9_H8", 4.86, 0.486, 4.19275, 3.635, 0.01424488212189472]
["eNOE-U6_H2';G9_H1", 3.55, 0.355, 3.8607499999999995, 3.528, 0.11957807770100123]
["eNOE-U6_H2';C8_H41", 3.73, 0.373, 3.55775, 3.6430000000000002, 0.23186687991172844]
["eNOE-C8_H4';G9_H8", 5.48, 0.5479999999999999, 4.89, 4.827, 0.06873621073834854]
['eNOE-C8_H5";G9_H8', 5.17, 0.517, 5.1282499999999995, 4.864, 0.07443733382293226]
["eNOE-C8_H4';G10_H8", 5.77, 0.5770000000000001, 6.1282499999999995, 6.893, 0.6632723799465801]
['eNOE-C13_H42;C13_H5', 2.62, 0.262, 2.4415000000000004, 2.4290000000000003, 0.055824128594482575]
```

```
# number of training examples: 40000
# number of test     examples: 10000
# number of input features:    251
# Training accuracy 0.998 ((40000, 251))
# Test     accuracy 0.998 ((10000, 251))
# Training F1 score 0.999 ((40000, 251))
# Test     F1 score 0.999 ((10000, 251))
   0  126        U6_H2';C8_H42 1.0000 
   1   80         U6_H3';C8_H5 0.8332 
   2   91         U6_H6;C8_H42 0.8275 
   3  151         C8_H42;G9_H1 0.7401 
   4  150         C8_H1';G9_H1 0.6925 
   5  122        U6_H2';C8_H41 0.6240 
   6  133         C8_H5;U7_H5' 0.6221 
   7  127        U6_H3';C8_H42 0.6062 
   8  146         U6_H1';G9_H1 0.5761 
   9  124         U6_H5;C8_H41 0.5296 
  10  123        U6_H3';C8_H41 0.5142 
  11  145         G9_H1;G10_H8 0.4697 
  12  148        U6_HO2';G9_H1 0.3784 
  13  147         U6_H2';G9_H1 0.3584 
  14  137         U6_H2';C8_H6 0.3430 
  15  134        C8_H5;U7_H5'' 0.3384 
  16  158        C8_H4';G9_H5' 0.3263 
  17  128       C8_H42;U7_H5'' 0.2107 
  18  132         C8_H5;U7_H3' 0.1634 
  19  178         G9_H1;G10_H1 0.1589 
  20  243       C14_H42;C14_H5 0.1478 
       U6_H2';C8_H42 3.12 3.44 2.96 8.69 2.92
        U6_H3';C8_H5 3.27 4.40 3.93 8.64 3.79
        U6_H6;C8_H42 4.07 4.85 4.24 10.86 4.28
        C8_H42;G9_H1 3.94 6.62 5.82 14.34 5.88
        C8_H1';G9_H1 3.56 4.52 3.94 10.47 4.04
       U6_H2';C8_H41 3.20 4.26 3.75 9.24 3.65
        C8_H5;U7_H5' 3.45 4.35 3.97 7.41 3.68
       U6_H3';C8_H42 2.78 3.71 3.16 10.09 2.60
        U6_H1';G9_H1 2.92 4.15 3.41 14.38 3.46
        U6_H5;C8_H41 4.33 4.53 3.86 12.22 3.68
       U6_H3';C8_H41 2.96 4.61 4.02 10.68 3.52
        G9_H1;G10_H8 4.43 6.32 5.84 10.07 5.73
       U6_HO2';G9_H1 2.82 4.10 3.37 14.31 3.08
        U6_H2';G9_H1 3.14 4.60 3.84 14.32 3.54
        U6_H2';C8_H6 3.49 4.18 3.86 6.73 3.95
       C8_H5;U7_H5'' 2.29 3.10 2.71 7.07 2.35
       C8_H4';G9_H5' 4.43 4.08 3.92 5.08 4.04
      C8_H42;U7_H5'' 4.98 4.95 4.45 9.38 4.21
["U6_H2';C8_H42", "U6_H3';C8_H5", 'U6_H6;C8_H42', 'C8_H42;G9_H1', "C8_H1';G9_H1", "U6_H2';C8_H41", "C8_H5;U7_H5'", "U6_H3';C8_H42", "U6_H1';G9_H1", 'U6_H5;C8_H41', "U6_H3';C8_H41", 'G9_H1;G10_H8', "U6_HO2';G9_H1", "U6_H2';G9_H1", "U6_H2';C8_H6", "C8_H5;U7_H5''", "C8_H4';G9_H5'", "C8_H42;U7_H5''"]
["NOE-U6_H2';C8_H42", 3.12, 0.97, 3.016, 2.89, 0.4778137712540317]
["NOE-U6_H3';C8_H5", 3.27, 1.07, 3.91725, 3.78, 0.06389248782133927]
['NOE-U6_H6;C8_H42', 4.07, 2.07, 4.28125, 4.263999999999999, 0.21838555355151132]
['NOE-C8_H42;G9_H1', 3.94, 1.94, 5.871499999999999, 5.869, 0.20803925911551738]
["NOE-C8_H1';G9_H1", 3.56, 1.26, 3.98625, 4.004, 0.15622713165985805]
["NOE-U6_H2';C8_H41", 3.2, 1.28, 3.55775, 3.645, 0.23186687991172844]
["NOE-C8_H5;U7_H5'", 3.45, 1.19, 3.92175, 3.677, 0.3759551170020167]
["NOE-U6_H3';C8_H42", 2.78, 0.77, 3.15975, 2.588, 0.38695079359870405]
["NOE-U6_H1';G9_H1", 2.92, 0.85, 3.38975, 3.444, 0.16839314119048926]
['NOE-U6_H5;C8_H41', 4.33, 2.255, 3.821, 3.6489999999999996, 0.3395987828796405]
["NOE-U6_H3';C8_H41", 2.96, 0.88, 3.769, 3.511, 0.19424211695716273]
['NOE-G9_H1;G10_H8', 4.43, 2.26, 5.8375, 5.7170000000000005, 0.2182681836640422]
["NOE-U6_HO2';G9_H1", 2.82, 0.79, 3.371, 3.071, 0.10611314715905851]
["NOE-U6_H2';G9_H1", 3.14, 0.99, 3.8607499999999995, 3.5239999999999996, 0.11957807770100123]
["NOE-U6_H2';C8_H6", 3.49, 1.22, 3.80975, 3.949, 0.08020962120178181]
["NOE-C8_H5;U7_H5''", 2.29, 0.53, 2.6422499999999998, 2.343, 0.15463155132981982]
["NOE-C8_H4';G9_H5'", 4.43, 2.26, 3.83725, 4.022, 0.0967793883014353]
["NOE-C8_H42;U7_H5''", 4.98, 2.31, 4.553750000000001, 4.2, 0.6378685209351536]
```

```
# number of training examples: 40000
# number of test     examples: 10000
# number of input features:    96
# Training accuracy 0.948 ((40000, 96))
# Test     accuracy 0.946 ((10000, 96))
# Training F1 score 0.970 ((40000, 96))
# Test     F1 score 0.969 ((10000, 96))
   0   40               8-C4Pe 1.0000 
   1   89              C8-H3H4 0.4991 
   2   27              10-C4Pb 0.4313 
   3   68              C8-H1H2 0.3726 
   4   67              U7-H1H2 0.3021 
   5    7              10-1H5P 0.2876 
   6   69              G9-H1H2 0.2848 
   7   88              U7-H3H4 0.2683 
   8   59             G9-2H5H4 0.2524 
   9   49             G9-1H5H4 0.1951 
  10   90              G9-H3H4 0.1919 
  11   18              10-2H5P 0.1646 
  12   32                7-H3P 0.1377 
  13   33                8-H3P 0.0914 
  14   41               9-C4Pe 0.0696 
  15   25               8-C4Pb 0.0638 
  16   57             U7-2H5H4 0.0498 
  17   80              G9-H2H3 0.0431 
  18   60            U11-2H5H4 0.0309 
  19    4               7-1H5P 0.0293 
  20   34                9-H3P 0.0291 
              8-C4Pe 7.10 1.40 0.65 5.59 1.16
             C8-H3H4 0.60 1.64 1.04 5.04 0.29
             10-C4Pb 6.10 3.08 1.89 9.76 10.67
             C8-H1H2 9.30 10.15 10.62 7.48 10.99
             U7-H1H2 8.00 9.90 10.56 6.19 10.37
             10-1H5P 1.30 9.38 10.41 3.56 4.09
             G9-H1H2 0.60 2.36 2.07 4.02 1.53
             U7-H3H4 1.00 2.31 1.55 6.57 0.92
            G9-2H5H4 2.80 10.33 10.87 7.29 10.39
['8-C4Pe', 'C8-H3H4', '10-C4Pb', 'C8-H1H2', 'U7-H1H2', '10-1H5P', 'G9-H1H2', 'U7-H3H4', 'G9-2H5H4']
['J3-8-C4Pe', 7.1, 1.5, 1.3219999999999998, 1.165, 0.2314965802483196]
['J3-C8-H3H4', 0.6, 1.5, 1.565, 0.2871, 0.10819118879711664]
['J3-10-C4Pb', 6.1, 1.5, 2.964, 10.67, 1.2784266371859854]
['J3-C8-H1H2', 9.3, 1.5, 10.2655, 10.99, 0.2743981292453231]
['J3-U7-H1H2', 8.0, 1.5, 10.030999999999999, 10.37, 0.22725316279427216]
['J3-10-1H5P', 1.3, 1.5, 9.289000000000001, 4.092, 2.158626260688342]
['J3-G9-H1H2', 0.6, 1.5, 2.6267500000000004, 1.526, 0.816818778350906]
['J3-U7-H3H4', 1.0, 1.5, 2.158, 0.9209, 0.25088244259015013]
['J3-G9-2H5H4', 2.8, 1.5, 10.37425, 10.39, 0.5358783288521127]
```

```
# number of training examples: 40000
# number of test     examples: 10000
# number of input features:    84
# Training accuracy 0.985 ((40000, 84))
# Test     accuracy 0.985 ((10000, 84))
# Training F1 score 0.991 ((40000, 84))
# Test     F1 score 0.991 ((10000, 84))
   0    5             G9:C4p-P 1.0000 
   1   36        U7:C3p-P-plus 0.8532 
   2   26             G9:C5s-P 0.6034 
   3   66             U7:C3-C4 0.5707 
   4   67             C8:C3-C4 0.4956 
   5   15             G9:C5p-P 0.4284 
   6   56             C8:C1-C2 0.4099 
   7   55             U7:C1-C2 0.3495 
   8    3             U7:C4p-P 0.2882 
   9   78             G9:C1-CC 0.2437 
  10   57             G9:C1-C2 0.2407 
  11   14             C8:C5p-P 0.1746 
  12   68             G9:C3-C4 0.1443 
  13   24             U7:C5s-P 0.1252 
  14   37        C8:C3p-P-plus 0.0928 
  15   35        U6:C3p-P-plus 0.0654 
  16   25             C8:C5s-P 0.0483 
  17    4             C8:C4p-P 0.0381 
  18   13             U7:C5p-P 0.0254 
  19   16            G10:C5p-P 0.0223 
  20   17            U11:C5p-P 0.0174 
            G9:C4p-P 11.00 9.30 12.09 -6.37 16.03
       U7:C3p-P-plus 1.20 2.87 5.43 -11.51 11.51
            G9:C5s-P -0.40 -13.31 -15.73 0.28 -8.81
            U7:C3-C4 -4.50 -3.51 -5.35 6.84 -7.10
            C8:C3-C4 -7.00 -5.09 -6.55 3.15 -8.66
            G9:C5p-P 4.60 9.11 10.90 -0.99 11.35
            C8:C1-C2 17.30 15.49 16.65 8.98 17.81
            U7:C1-C2 15.80 14.88 16.49 5.84 16.36
            U7:C4p-P 15.10 12.48 13.98 4.08 15.08
            G9:C1-CC 17.00 15.73 16.68 10.38 17.89
            G9:C1-C2 -8.30 -3.47 -4.20 0.58 -5.11
['G9:C4p-P', 'U7:C3p-P-plus', 'G9:C5s-P', 'U7:C3-C4', 'C8:C3-C4', 'G9:C5p-P', 'C8:C1-C2', 'U7:C1-C2', 'U7:C4p-P', 'G9:C1-CC', 'G9:C1-C2']
['CCRR-G9:C4p-P', 11.0, 2.0, 7.7905, 13.08, 2.4071582831214076]
['CCRR-U7:C3p-P-plus', 1.2, 2.0, 2.2535, 9.373, 2.57671114666222]
['CCRR-G9:C5s-P', -0.4, 2.0, -10.60725, -7.185, 2.8242090780724194]
['CCRR-U7:C3-C4', -4.5, 2.0, -3.22375, -5.797000000000001, 0.49975352258221584]
['CCRR-C8:C3-C4', -7.0, 2.0, -4.395250000000001, -7.069, 0.26308347851331654]
['CCRR-G9:C5p-P', 4.6, 2.0, 7.52125, 9.244, 1.432755707253218]
['CCRR-C8:C1-C2', 17.3, 2.0, 13.1, 14.52, 0.6618660488850193]
['CCRR-U7:C1-C2', 15.8, 2.0, 12.627500000000001, 13.35, 0.4800260409602799]
['CCRR-U7:C4p-P', 15.1, 2.0, 10.3575, 12.3, 0.225]
['CCRR-G9:C1-CC', 17.0, 2.0, 13.149999999999999, 14.6, 0.5771770381203095]
['CCRR-G9:C1-C2', -8.3, 2.0, -2.3593125, -4.169, 1.6152768257974646]
```

```
# number of training examples: 40000
# number of test     examples: 10000
# number of input features:    32
# Training accuracy 0.968 ((40000, 32))
# Test     accuracy 0.967 ((10000, 32))
# Training F1 score 0.981 ((40000, 32))
# Test     F1 score 0.981 ((10000, 32))
   0    4        8.C.C6-8.C.H6 1.0000 
   1   12      10.G.C8-10.G.H8 0.9210 
   2    2        6.U.C6-6.U.H6 0.6715 
   3   29      10.G.N1-10.G.H1 0.3577 
   4   21      8.C.C1'-8.C.H1' 0.2596 
   5   22      9.G.C1'-9.G.H1' 0.2394 
   6   19      6.U.C1'-6.U.H1' 0.2218 
   7    9        2.G.C8-2.G.H8 0.1558 
   8   11        9.G.C8-9.G.H8 0.1452 
   9    5      11.U.C6-11.U.H6 0.1318 
  10    8        1.G.C8-1.G.H8 0.1085 
  11   25    13.C.C1'-13.C.H1' 0.0807 
  12   31      11.U.N3-11.U.H3 0.0605 
  13   24    12.G.C1'-12.G.H1' 0.0500 
  14   20      7.U.C1'-7.U.H1' 0.0461 
  15    1        5.C.C6-5.C.H6 0.0392 
  16   27        2.G.N1-2.G.H1 0.0294 
  17   18      5.C.C1'-5.C.H1' 0.0255 
  18    6      13.C.C6-13.C.H6 0.0243 
  19   26    14.C.C1'-14.C.H1' 0.0223 
  20   17      4.A.C1'-4.A.H1' 0.0141 
       8.C.C6-8.C.H6 -3.66 -7.29 -9.80 6.84 -3.15
     10.G.C8-10.G.H8 11.10 6.71 7.77 0.75 8.25
       6.U.C6-6.U.H6 4.00 6.71 7.15 4.22 3.62
     10.G.N1-10.G.H1 -3.88 2.53 3.04 -0.37 3.97
     8.C.C1'-8.C.H1' -14.66 -4.92 -5.82 0.14 -12.33
     9.G.C1'-9.G.H1' -5.14 -3.14 -3.95 1.41 -3.12
     6.U.C1'-6.U.H1' 6.89 11.57 11.92 9.59 9.94
['8.C.C6-8.C.H6', '10.G.C8-10.G.H8', '6.U.C6-6.U.H6', '10.G.N1-10.G.H1', "8.C.C1'-8.C.H1'", "9.G.C1'-9.G.H1'", "6.U.C1'-6.U.H1'"]
['RDC-8.C.C6-8.C.H6', -3.66, 1.0, -5.022749999999999, -2.0, 2.3807355130995407]
['RDC-10.G.C8-10.G.H8', 11.1, 1.0, 4.7855, 5.195, 0.7160551654726054]
['RDC-6.U.C6-6.U.H6', 4.0, 1.0, 4.74275, 2.275, 0.4385098820627271]
['RDC-10.G.N1-10.G.H1', -3.88, 1.0, 1.8345, 2.496, 0.29322857978034805]
["RDC-8.C.C1'-8.C.H1'", -14.66, 1.0, -3.1789, -7.767, 1.5646372231287355]
["RDC-9.G.C1'-9.G.H1'", -5.14, 1.0, -2.332, -1.9780000000000002, 0.5470655049138693]
["RDC-6.U.C1'-6.U.H1'", 6.89, 1.0, 8.17925, 6.252999999999999, 0.09658631718140359]
```

```
# number of training examples: 40000
# number of test     examples: 10000
# number of input features:    39
# Training accuracy 0.985 ((40000, 39))
# Test     accuracy 0.983 ((10000, 39))
# Training F1 score 0.991 ((40000, 39))
# Test     F1 score 0.990 ((10000, 39))
   0   19      8.C.C4'-8.C.H4' 1.0000 
   1   21        8.C.C6-8.C.H6 0.9119 
   2   23      8.C.C3'-8.C.H3' 0.6199 
   3   25      9.G.C3'-9.G.H3' 0.3662 
   4   14        6.U.C6-6.U.H6 0.3022 
   5   24      9.G.C1'-9.G.H1' 0.2269 
   6   20      8.C.C1'-8.C.H1' 0.1622 
   7   29      11.U.C5-11.U.H5 0.1581 
   8   13      6.U.C1'-6.U.H1' 0.1482 
   9   10        5.C.C6-5.C.H6 0.1164 
  10   32    13.C.C1'-13.C.H1' 0.1020 
  11    3        2.G.C8-2.G.H8 0.0875 
  12   12      5.C.C2'-5.C.H2' 0.0869 
  13   16      7.U.C4'-7.U.H4' 0.0764 
  14   38      14.C.C6-14.C.H6 0.0447 
  15   28      11.U.C6-11.U.H6 0.0372 
  16    4        3.C.C5-3.C.H5 0.0312 
  17    1        1.G.C8-1.G.H8 0.0310 
  18   30    12.G.C1'-12.G.H1' 0.0275 
  19   36    14.C.C4'-14.C.H4' 0.0214 
  20    9      5.C.C1'-5.C.H1' 0.0150 
     8.C.C4'-8.C.H4' 3.80 8.45 11.07 -6.24 6.49
       8.C.C6-8.C.H6 -4.86 -7.29 -9.80 6.84 -3.15
     8.C.C3'-8.C.H3' -14.78 -10.67 -12.24 -1.87 -14.05
     9.G.C3'-9.G.H3' 10.20 -1.72 -2.59 3.20 4.18
       6.U.C6-6.U.H6 6.13 6.71 7.15 4.22 3.62
     9.G.C1'-9.G.H1' -5.07 -3.14 -3.95 1.41 -3.12
["8.C.C4'-8.C.H4'", '8.C.C6-8.C.H6', "8.C.C3'-8.C.H3'", "9.G.C3'-9.G.H3'", '6.U.C6-6.U.H6', "9.G.C1'-9.G.H1'"]
["RDC1-8.C.C4'-8.C.H4'", 3.8, 1.4, 8.45975, 7.013999999999999, 2.954669115710477]
['RDC1-8.C.C6-8.C.H6', -4.86, 1.4, -7.28225, -3.444, 3.444418226154697]
["RDC1-8.C.C3'-8.C.H3'", -14.78, 1.4, -10.966750000000001, -15.21, 2.3313534516813763]
["RDC1-9.G.C3'-9.G.H3'", 10.2, 1.4, -1.7861500000000001, 4.553999999999999, 0.9655093077403933]
['RDC1-6.U.C6-6.U.H6', 6.13, 1.4, 6.88625, 3.928, 0.6005122119213451]
["RDC1-9.G.C1'-9.G.H1'", -5.07, 1.4, -3.3902499999999995, -3.4160000000000004, 0.804811934553657]
```

```
# number of training examples: 40000
# number of test     examples: 10000
# number of input features:    14
# Training accuracy 0.967 ((40000, 14))
# Test     accuracy 0.967 ((10000, 14))
# Training F1 score 0.981 ((40000, 14))
# Test     F1 score 0.981 ((10000, 14))
   0    8      10.G.C8-10.G.H8 1.0000 
   1    7      9.G.C2'-9.G.H2' 0.4695 
   2   13      7.U.C2'-7.U.H2' 0.3491 
   3   12    10.G.C3'-10.G.H3' 0.2326 
   4    9    11.U.C3'-11.U.H3' 0.1564 
   5    4      6.U.C2'-6.U.H2' 0.1422 
   6   11      6.U.C4'-6.U.H4' 0.1231 
   7    3      4.A.C1'-4.A.H1' 0.1073 
   8    0      1.G.C1'-1.G.H1' 0.0442 
   9    5        7.U.C6-7.U.H6 0.0085 
  10    2      2.G.C4'-2.G.H4' 0.0079 
  11    6        7.U.C5-7.U.H5 0.0067 
  12    1      1.G.C2'-1.G.H2' 0.0033 
  13   10      14.C.C5-14.C.H5 0.0011 
     10.G.C8-10.G.H8 14.76 6.71 7.77 0.75 8.25
     9.G.C2'-9.G.H2' 2.64 -4.73 -5.54 -0.18 -4.02
     7.U.C2'-7.U.H2' -1.29 -8.83 -10.08 -1.82 -11.73
   10.G.C3'-10.G.H3' -11.37 -9.39 -10.23 -4.67 -6.57
['10.G.C8-10.G.H8', "9.G.C2'-9.G.H2'", "7.U.C2'-7.U.H2'", "10.G.C3'-10.G.H3'"]
['RDC2-10.G.C8-10.G.H8', 14.76, 2.2, 3.97075, 4.9639999999999995, 0.5278809051291778]
["RDC2-9.G.C2'-9.G.H2'", 2.64, 2.2, -2.8652500000000005, -2.4130000000000003, 0.5120028482993688]
["RDC2-7.U.C2'-7.U.H2'", -1.29, 2.2, -5.055, -7.053, 0.8074730129649326]
["RDC2-10.G.C3'-10.G.H3'", -11.37, 2.2, -5.621, -3.9560000000000004, 0.4475079142689363]
```

```
# number of training examples: 40000
# number of test     examples: 10000
# number of input features:    91
# Training accuracy 0.992 ((40000, 91))
# Test     accuracy 0.992 ((10000, 91))
# Training F1 score 0.995 ((40000, 91))
# Test     F1 score 0.995 ((10000, 91))
   0   42             8Cyt1H2' 1.0000 
   1   43              8CytH3' 0.7939 
   2   50               9GuaH1 0.6516 
   3   62             10GuaH3' 0.6360 
   4   63            10Gua1H5' 0.6303 
   5   52             9Gua1H2' 0.5709 
   6   28             6Ura1H2' 0.5065 
   7   65              10GuaH8 0.4949 
   8   44              8CytH4' 0.3296 
   9   41              8CytH1' 0.3135 
  10   53              9GuaH3' 0.2892 
  11   56               9GuaH8 0.2589 
  12   29               6UraH3 0.2274 
  13   35             7Ura1H2' 0.1956 
  14   45              8CytH41 0.1872 
  15   46              8CytH42 0.1804 
  16   36              7UraH4' 0.1711 
  17   48             8Cyt1H5' 0.1527 
  18   34              7UraH1' 0.1226 
  19   54             9Gua1H5' 0.1014 
  20   47               8CytH5 0.0833 
            8Cyt1H2' 0.40 77.46 82.39 49.75 83.32
             8CytH3' 0.44 85.18 90.52 55.16 83.36
              9GuaH1 0.45 46.31 24.66 167.99 23.63
            10GuaH3' 0.23 53.36 55.36 42.13 48.85
           10Gua1H5' 0.37 102.92 109.70 64.80 98.14
            9Gua1H2' 0.37 56.93 53.11 78.35 53.71
            6Ura1H2' 0.15 32.26 31.06 38.98 30.10
             10GuaH8 0.19 70.12 75.37 40.62 45.97
             8CytH4' 0.31 51.50 46.60 79.01 44.08
             8CytH1' 0.28 52.15 48.64 71.85 52.70
             9GuaH3' 0.32 47.36 45.76 56.39 47.31
              9GuaH8 0.96 132.52 129.84 147.58 117.14
              6UraH3 2.36 45.56 42.62 62.09 44.05
["8Cyt1H2'", "8CytH3'", '9GuaH1', "10GuaH3'", "10Gua1H5'", "9Gua1H2'", "6Ura1H2'", '10GuaH8', "8CytH4'", "8CytH1'", "9GuaH3'", '9GuaH8', '6UraH3']
["sPRE-8Cyt1H2'", 0.399957834, 0.023342317999999997, 0.473025, 0.4939, 0.05201354791462188]
["sPRE-8CytH3'", 0.444052131, 0.012539368, 0.520025, 0.5225, 0.04060668048486604]
['sPRE-9GuaH1', 0.44688911299999995, 0.041625286000000004, 0.286775, 0.1447, 0.14133721791045223]
["sPRE-10GuaH3'", 0.22554771199999998, 0.007794405999999999, 0.32994999999999997, 0.2964, 0.01691715106038838]
["sPRE-10Gua1H5'", 0.36991837200000005, 0.014758638999999999, 0.6353249999999999, 0.5896, 0.05774607490268636]
["sPRE-9Gua1H2'", 0.36606826200000003, 0.014027025, 0.35200000000000004, 0.3262, 0.03060620852049467]
["sPRE-6Ura1H2'", 0.15311677699999998, 0.0057467640000000006, 0.19955, 0.1849, 0.0043516280478306845]
['sPRE-10GuaH8', 0.185958138, 0.013338631, 0.43375, 0.2753, 0.03560903443416199]
["sPRE-8CytH4'", 0.305415241, 0.005661006999999999, 0.32877500000000004, 0.2699, 0.07280752593882953]
["sPRE-8CytH1'", 0.28496858, 0.009647694, 0.329575, 0.3294, 0.036552371104120344]
["sPRE-9GuaH3'", 0.315390596, 0.008019003, 0.2963, 0.2851, 0.01979141227906691]
['sPRE-9GuaH8', 0.960143912, 0.09971114699999999, 0.82175, 0.7235, 0.04537528695960684]
['sPRE-6UraH3', 2.361771788, 0.237358006, 0.28565, 0.2773, 0.015629992535720116]
```

To understand whether our MD+eNOE ensemble better reflects the loop dynamics, we make a scatter plot showing the Z-score of 2KOC vs the Z-score for MD+enoe. Points below the diagonal indicate that 2KOC has a lower error than MD+eNOE

In [13]:

```
A1 = ["eNOE_unidir","eNOE"]
A2 = ["RDC","NOE","J3","CCRR"]
A3 = ["RDC1","RDC2"]
A4 = ["sPRE"]
suspects2 = []
labels2 = []
classes = {"eNOE_unidir":"^","eNOE":"o","NOE":"^","J3":"o","RDC":"s","RDC1":"^","RDC2":"o","sPRE":"^","CCRR":"*"}
classes_c = {"eNOE_unidir":"k","eNOE":"0.7","NOE":"k","J3":"0.7","RDC":"0.2","RDC1":"k","RDC2":"0.7","sPRE":"k","CCRR":"0.5"}
legends = {"eNOE_unidir":"u-eNOE","eNOE":"eNOE","NOE":"NOE","J3":"3J couplings","RDC":"RDC","RDC1":"RDC1",\
           "RDC2":"RDC2","sPRE":"sPRE","CCRR":"CCRR"}

legends_lab = [0]*len(legends)
legends_name = list(legends.keys())

leg = []
cols = []
for j in range(len(suspects)):
    suspects2.append(suspects[j][1:])
    labels2.append(suspects[j][0])
    leg.append(classes[suspects[j][0].split("-")[0]])
    cols.append(classes_c[suspects[j][0].split("-")[0]])
    #print(labels2[-1])
suspects2 = np.array(suspects2)
#yy = np.arange(len(suspects2))

x = np.abs((suspects2[:,0]-suspects2[:,2]))/suspects2[:,1]
y = np.abs((suspects2[:,0]-suspects2[:,3]))/suspects2[:,1]
cha = [str(e) for e in range(100)]
nnu = 1
selection = ["eNOE-C8_H5';G9_H8","J3-10-1H5P","CCRR-U7:C3p-P-plus","RDC-8.C.C1'-8.C.H1'","RDC1-8.C.C3'-8.C.H3'","RDC2-10.G.C8-10.G.H8",\
            "sPRE-10Gua1H5'","sPRE-9GuaH1"]

selection_data = []
fig, ax = plt.subplots(2,2,figsize=(8,8))
plt.subplots_adjust(left=0.1, bottom=0.07, right=0.95, top=0.95, wspace=0.15, hspace=0.15)
pdb_best =0 
for u in range(len(x)):
    #print(labels2[u])
        
    if(labels2[u].split("-")[0] in A1): i1,i2,ln = 0,0,len(A1)
    if(labels2[u].split("-")[0] in A2): i1,i2,ln = 0,1,len(A2)
    if(labels2[u].split("-")[0] in A3): i1,i2,ln = 1,0,len(A3)
    if(labels2[u].split("-")[0] in A4): i1,i2,ln = 1,1,len(A4)
    if(x[u]>y[u]): pdb_best +=1
    if(legends_lab[legends_name.index(labels2[u].split("-")[0])]==0):
        ax[i1,i2].scatter(x[u],y[u],marker=leg[u],color=cols[u],label=legends[labels2[u].split("-")[0]])
        legends_lab[legends_name.index(labels2[u].split("-")[0])] += 1
    else:
        ax[i1,i2].scatter(x[u],y[u],marker=leg[u],color=cols[u])
    if(labels2[u] in selection):
        print(suspects2[u,:],labels2[u],x[u],y[u])

        ax[i1,i2].text(x[u],y[u],cha[nnu],ha="center",va="bottom",fontsize=13,color="r")
        selection_data.append([suspects2[u,0],suspects2[u,1],suspects2[u,2],suspects2[u,4],suspects2[u,3]])
        nnu += 1
print(pdb_best,len(x))


lims = [3.0,14,6,20]
k = 0 
for i1 in range(2):
    for i2 in range(2):
        ax[i1,i2].plot([-1,22],[-1,22],ls="--",c='0.25')
        ax[i1,i2].set_xlim(-0.1,lims[k])
        ax[i1,i2].set_ylim(-0.1,lims[k])
        ax[i1,i2].xaxis.set_major_locator(plt.MaxNLocator(4))
        ax[i1,i2].yaxis.set_major_locator(plt.MaxNLocator(4))

        #ax[i1,i2].set_xticks(4)
        #ax[i1,i2].set_yticks(4)
        k += 1
ax[0,0].set_ylabel("|Z-score| 2KOC")
ax[1,0].set_ylabel("|Z-score| 2KOC")
ax[1,0].set_xlabel("|Z-score| MD+set A")
ax[1,1].set_xlabel("|Z-score| MD+set A")
ax[0,0].set_title("set A")
ax[0,1].set_title("set B")
ax[1,0].set_title("set C")
ax[1,1].set_title("set D")
ax[0,0].legend()
ax[0,1].legend()
ax[1,0].legend()
ax[1,1].legend()
plt.savefig("figures/figure5.pdf")
plt.savefig("figures/figure5.png",dpi=600)
plt.close()
#plt.show()
#print(x.mean(),y.mean())
```

```
[4.86       0.486      4.19275    3.635      0.01424488] eNOE-C8_H5';G9_H8 1.372942386831276 2.520576131687244
[1.3        1.5        9.289      4.092      2.15862626] J3-10-1H5P 5.326000000000001 1.8613333333333333
[1.2        2.         2.2535     9.373      2.57671115] CCRR-U7:C3p-P-plus 0.5267499999999999 4.0865
[-14.66         1.          -3.1789      -7.767        1.56463722] RDC-8.C.C1'-8.C.H1' 11.4811 6.893
[-14.78         1.4        -10.96675    -15.21         2.33135345] RDC1-8.C.C3'-8.C.H3' 2.723749999999999 0.3071428571428582
[14.76        2.2         3.97075     4.964       0.52788091] RDC2-10.G.C8-10.G.H8 4.904204545454545 4.452727272727272
[0.44688911 0.04162529 0.286775   0.1447     0.14133722] sPRE-9GuaH1 3.8465588680880156 7.259748629715119
[0.36991837 0.01475864 0.635325   0.5896     0.05774607] sPRE-10Gua1H5' 17.983137062977143 14.884951654417456
56 95
```

In [14]:

```
%matplotlib inline
import matplotlib
import matplotlib.pyplot as plt
    
selection_name = ["eNOE, C8H5-G9H8 [$\AA$]","J3 G10 H5'-P [Hz]","CCRR, U7 C3'-C8 P [Hz]","RDC, C8 C1'-H1 [Hz]' ","RDC1, C8 C3'-H3' [Hz]",\
                  "RDC2, G10 C8-H8 [Hz]","sPRE, G10 H5' [AU]","sPRE, G9 H1 [AU]"]
print(len(selection_data))
fig, ax = plt.subplots(4,2,figsize=(7,4))
l = 0
plt.subplots_adjust(left=0.07, bottom=0.12, right=0.95, top=0.85, wspace=0.2, hspace=1.5)
for i in range(4):
    for k in range(2):
        ax[i,k].errorbar(selection_data[l][0],0.5,xerr=selection_data[l][1],fmt="s-",c="0.2",label="Expt")
        #print(selection_data[i])
        ax[i,k].errorbar(selection_data[l][2],0.5,xerr=selection_data[l][3],fmt="s-",label="MD+set A",c="#2c6fbb")
        if(l==1):
            ax[i,k].scatter(selection_data[l][4],0.5,c="orange",marker="s",label="2KOC")
        else:
            ax[i,k].scatter(selection_data[l][4],0.5,c="orange",marker="s",label="2KOC")
        if(i==0 and k == 0): 
            ax[i,k].legend(ncol=3,loc=1,bbox_to_anchor=(1.75,2.2))
        ax[i,k].set_yticks([])
    #ax[i].set_yticklabels(str(i+1),color="r",fontsize=12)
        ax[i,k].spines["top"].set_visible(False)
        ax[i,k].spines["right"].set_visible(False)
        #ax[i,k].spines["left"].set_visible(False)
        xm = ax[i,k].get_xlim()[0]
        xr = np.abs(ax[i,k].get_xlim()[1]-xm)
        ax[i,k].text(xm-0.1*xr,0.5,"%s" % (str(l+1)),va="center",ha="left",color="r",fontsize=10)
        ax[i,k].set_xlabel(selection_name[l])
    #ax[i].spines["left"].set_visible(False)

        l += 1
        
plt.savefig("figures/figure6.pdf")
plt.savefig("figures/figure6.png",dpi=600)
plt.show()
plt.close()
```

```
8
```

In [ ]:

```

```
