# Supplementary material for: Integrating NMR and simulations reveals motions in the UUCG tetraloop
Source: Nucleic Acids Res. 2020 May 19;48(11):5839–48. doi: 10.1093/nar/gkaa399 (PMC7293013; doi:10.1093/nar/gkaa399)
Supplement: gkaa399_Supplemental_Files [file gkaa399_supplemental_files.zip › SI6_comparison.pdf]

set_A eNOE

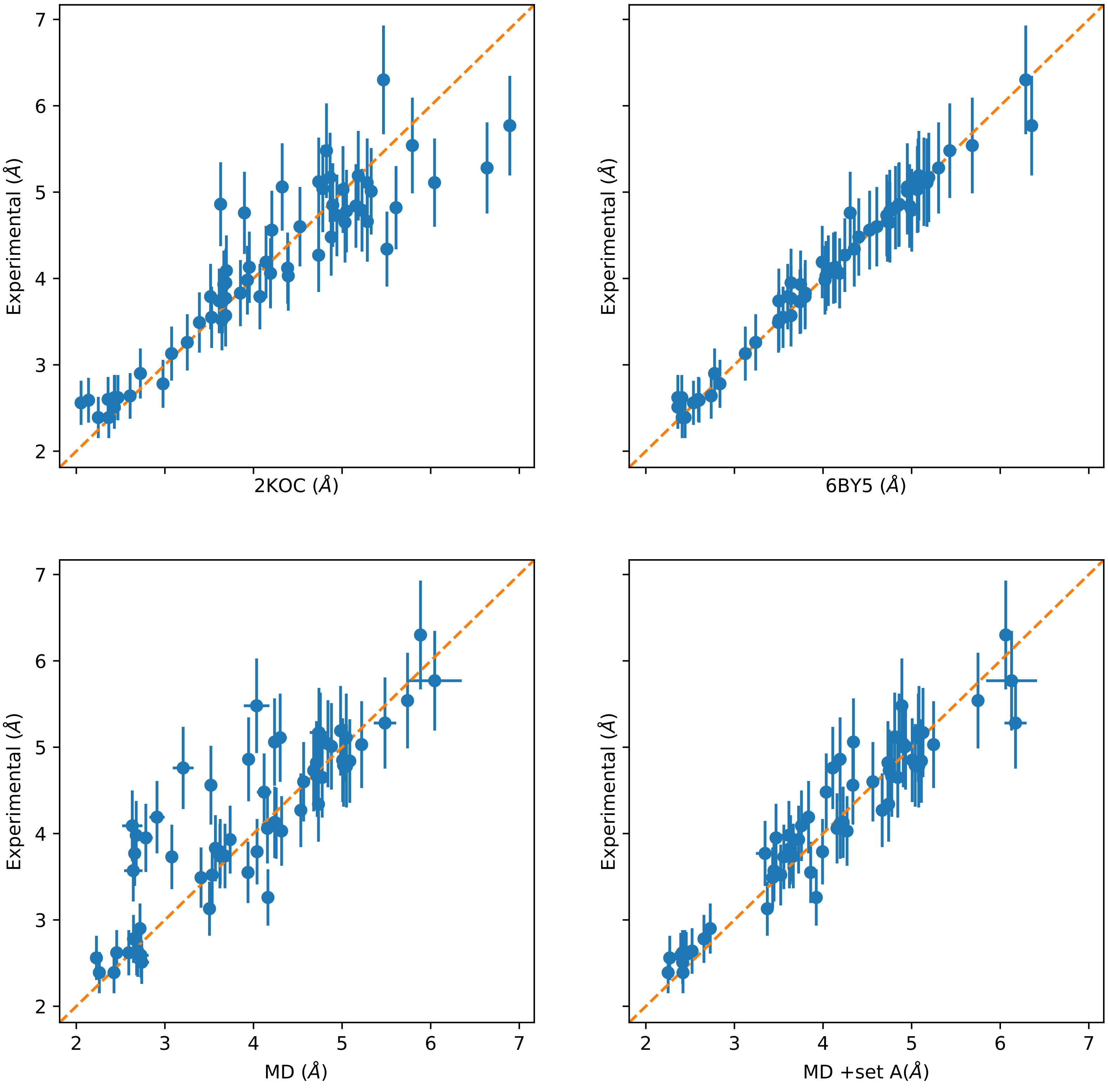

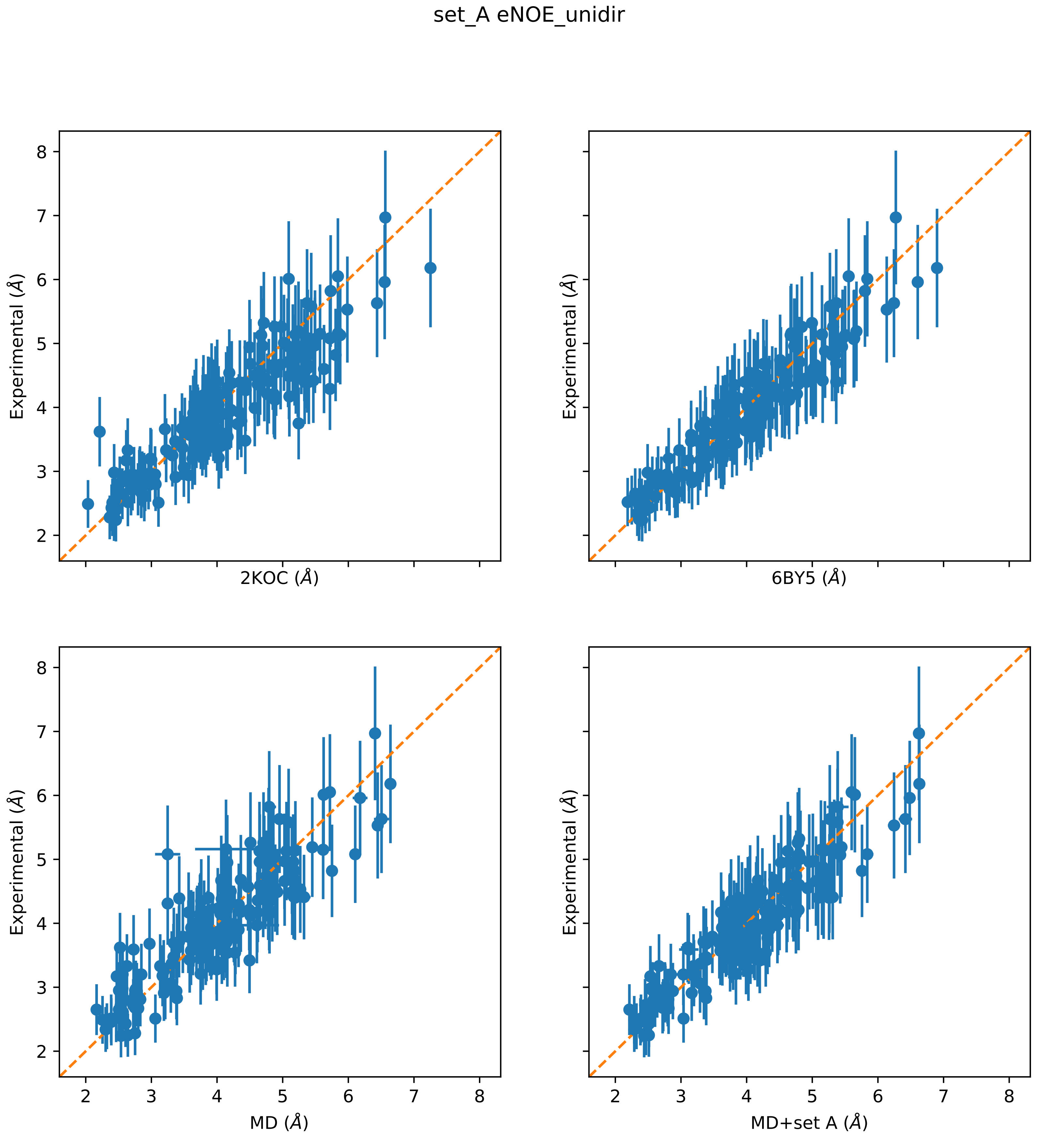

set_A eNOE_unidir
Experimental (Å)
2KOC (Å)
6BY5 (Å)
MD (Å)
MD+set A (Å)
2
3
4
5
6
7
8

set_A gn_eNOE, lower bound distances

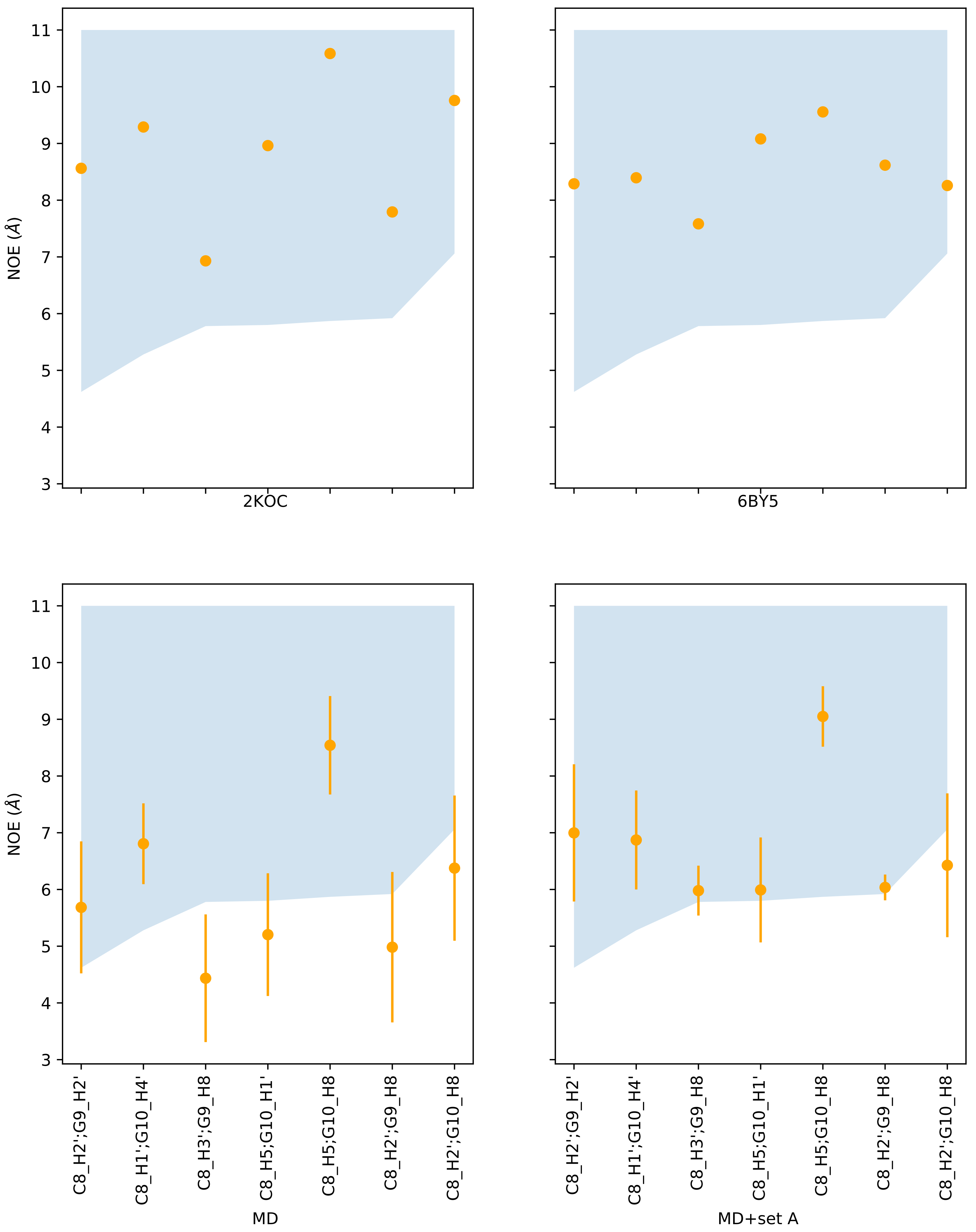

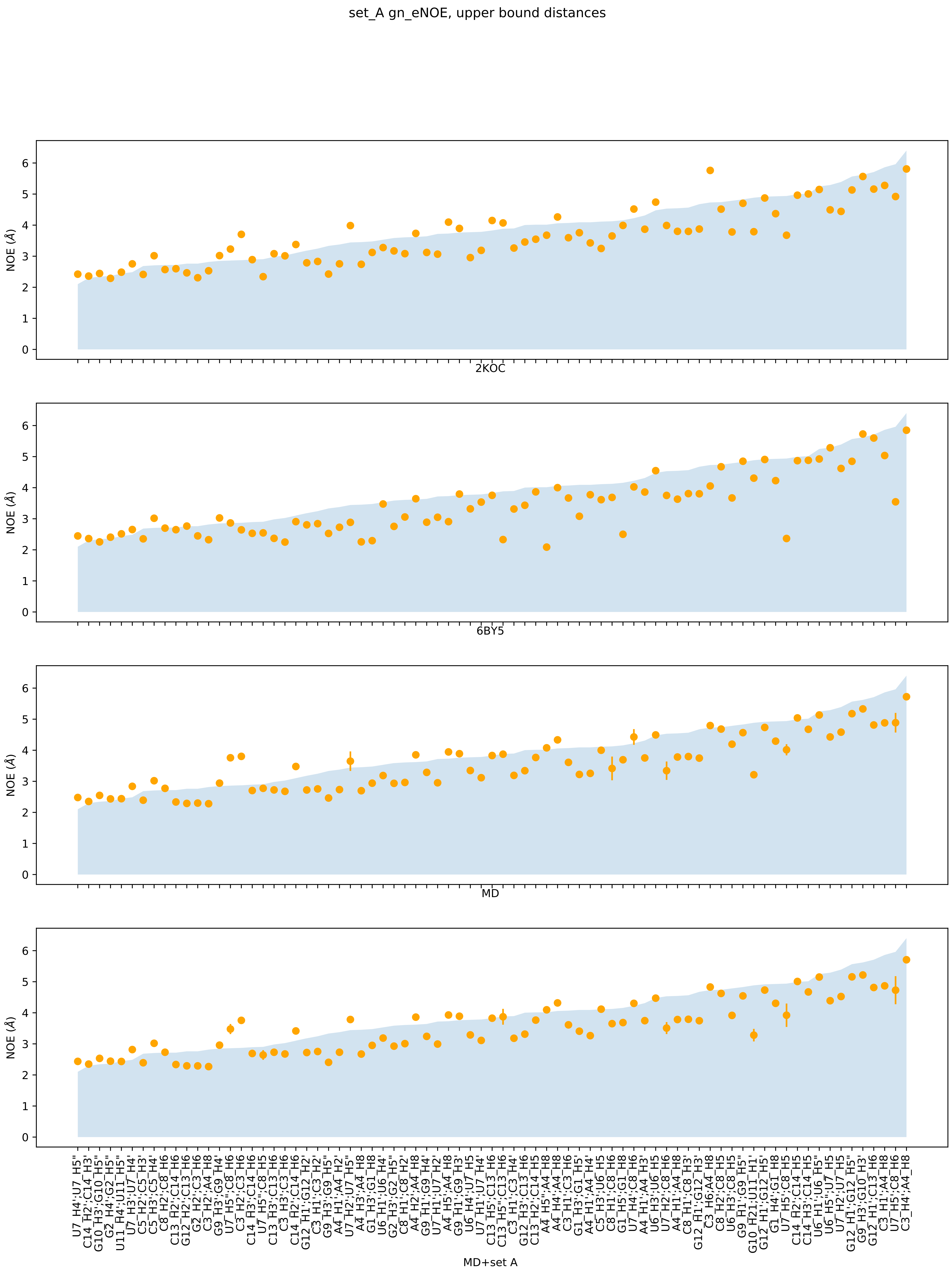

set_A gn_eNOE, upper bound distances
NOE (Å)
0
1
2
3
4
5
6
2KOC
6BY5
MD
MD+set A
U7_H4';U7_H5''
C14_H2';C14_H3'
G10_H3';G10_H5''
G2_H4';G2_H5''
U11_H4';U11_H5''
U7_H3';U7_H4'
C5_H2';C5_H3'
C5_H3';C5_H4'
C8_H2';C8_H6
C13_H2';C14_H6
G12_H2';C13_H6
G2_H2';C3_H6
C3_H2';A4_H8
G9_H3';G9_H4'
U7_H5'';C8_H6
C3_H2';C3_H6
C14_H3';C14_H6
U7_H5'';C8_H5
C13_H3';C13_H6
C3_H3';C3_H6
C14_H2';C14_H6
G12_H1';G12_H2'
C3_H1';C3_H2'
G9_H3';G9_H5''
A4_H1';A4_H2'
U7_H2';U7_H5''
A4_H3';A4_H8
G1_H3';G1_H8
U6_H1';U6_H4'
G2_H3';G2_H5''
C8_H1';C8_H2'
A4_H2';A4_H8
G9_H1';G9_H4'
U7_H1';U7_H2'
A4_H5';A4_H8
G9_H1';G9_H3'
U6_H4';U7_H5
U7_H1';U7_H4'
C13_H5';C13_H6
C13_H5'';C13_H6
C3_H1';C3_H4'
G12_H3';C13_H6
C13_H2';C14_H5
A4_H5'';A4_H8
A4_H4';A4_H8
C3_H1';C3_H6
G1_H3';G1_H5'
A4_H1';A4_H4'
C5_H3';U6_H5
C8_H1';C8_H6
G1_H5';G1_H8
U7_H4';C8_H6
A4_H1';A4_H3'
U6_H3';U6_H5
U7_H2';C8_H6
A4_H1';A4_H8
C8_H1';C8_H3'
G12_H1';G12_H3'
C3_H6;A4_H8
C8_H2';C8_H5
U6_H3';C8_H5
G9_H1';G9_H5''
G10_H21;U11_H1'
G12_H1';G12_H5'
G1_H4';G1_H8
U7_H5';C8_H5
C14_H2';C14_H5
C14_H3';C14_H5
U6_H1';U6_H5''
U6_H5'';U7_H5
U7_H2';U7_H5
G12_H1';G12_H5''
G9_H3';G10_H3'
G12_H1';C13_H6
C3_H1';A4_H8
U7_H5';C8_H6
C3_H4';A4_H8

set_B NOE

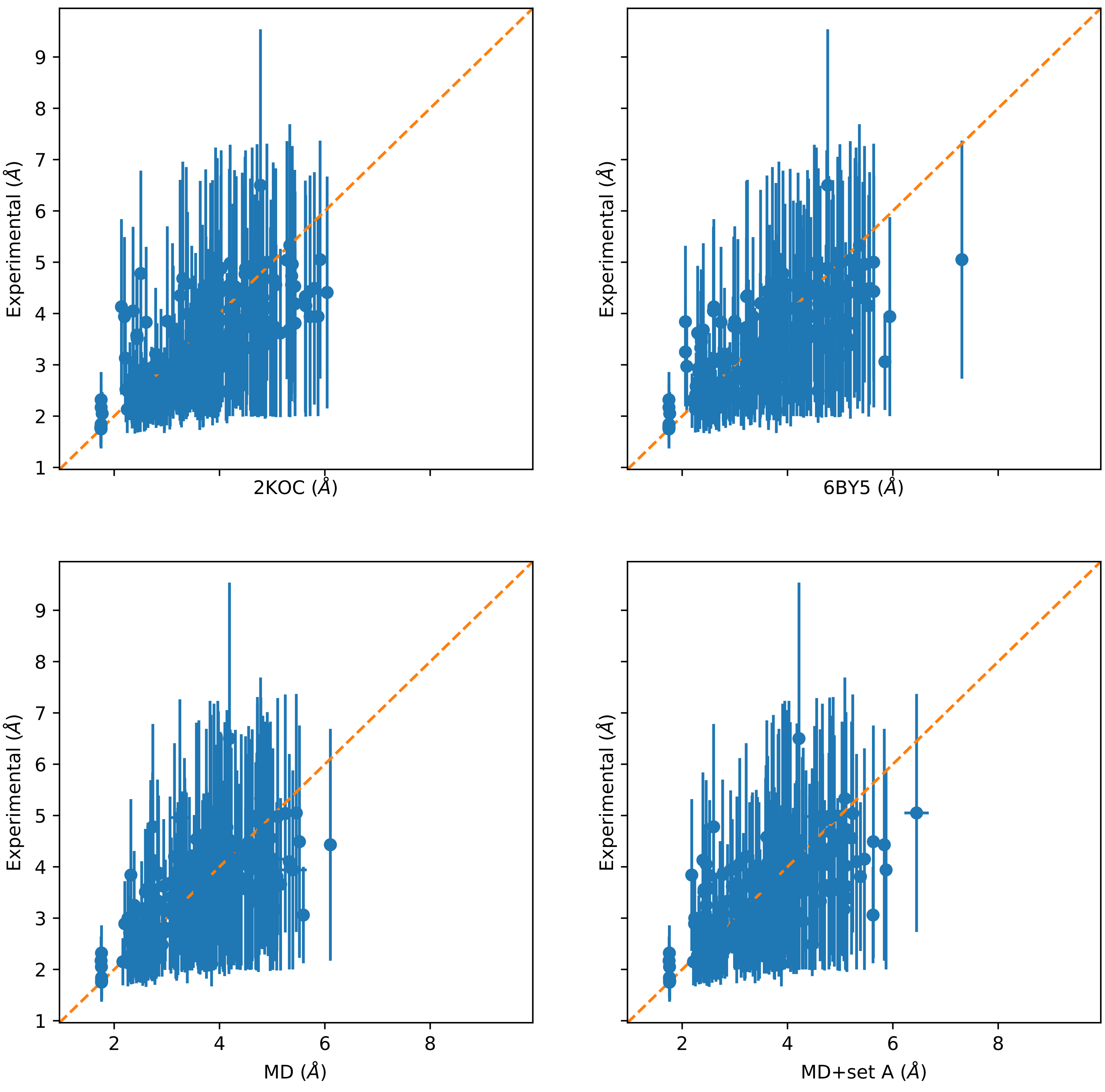

set_B RDC

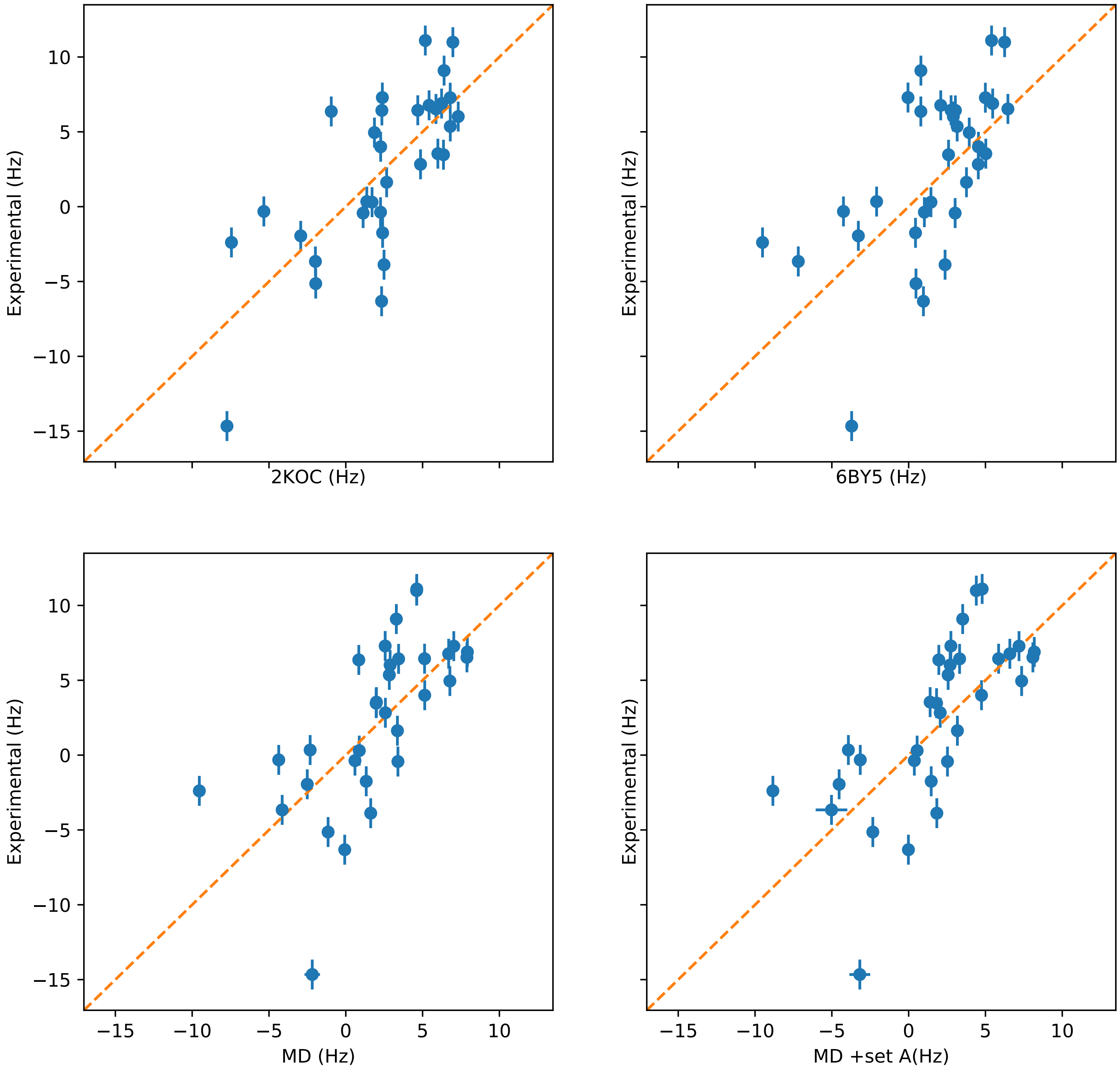

Experimental (Hz)
2KOC (Hz)
6BY5 (Hz)
MD (Hz)
MD +set A(Hz)
10
5
0
−5
−10
−15
−15
−10
−5
0
5
10

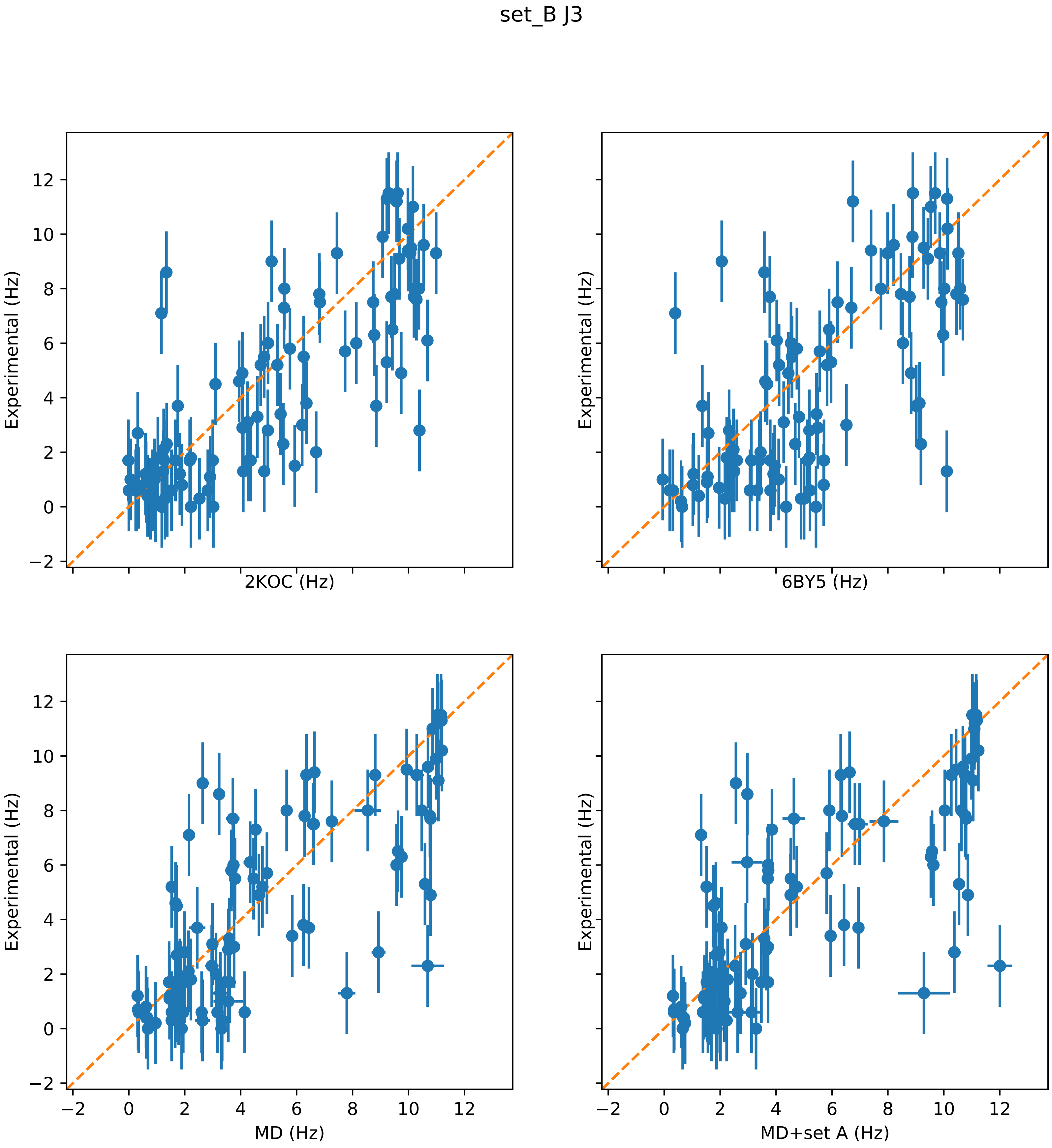
set_B J3
Experimental (Hz)
2KOC (Hz)
6BY5 (Hz)
MD (Hz)
MD+set A (Hz)
12
10
8
6
4
2
0
−2

set_B CCRR

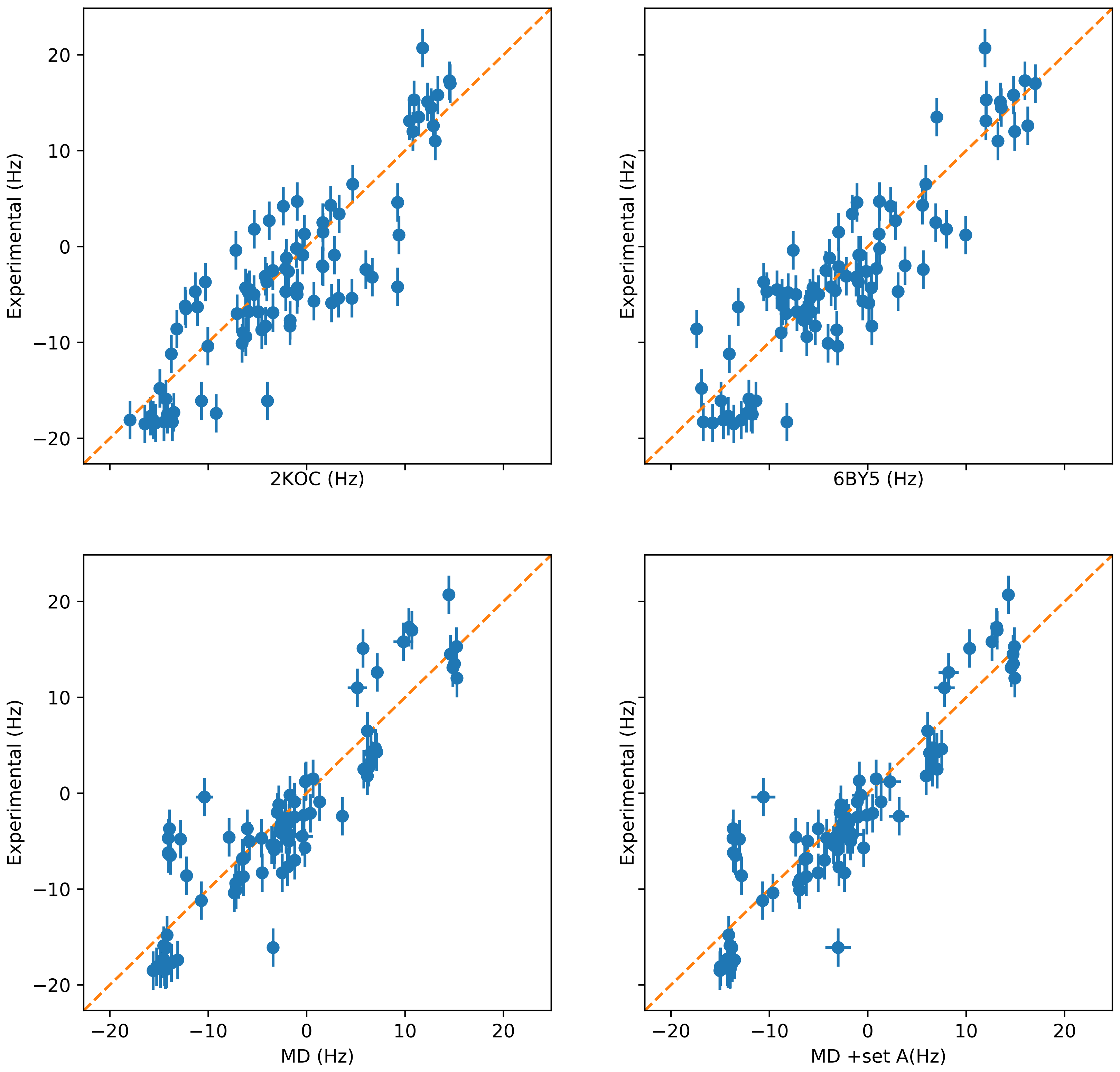

set_C RDC1

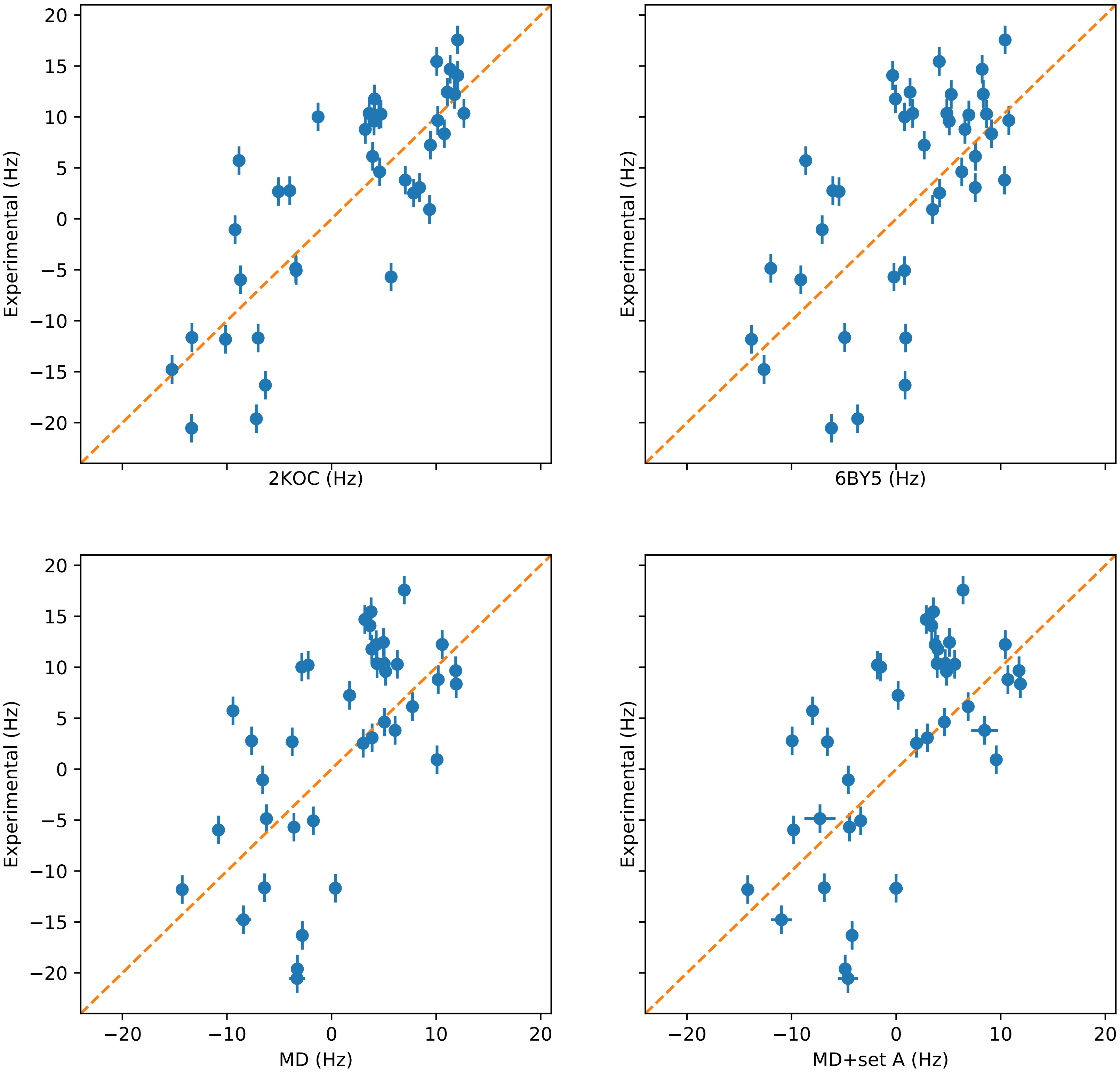

Experimental (Hz)
2KOC (Hz)
6BY5 (Hz)
MD (Hz)
MD+set A (Hz)
20
15
10
5
0
−5
−10
−15
−20

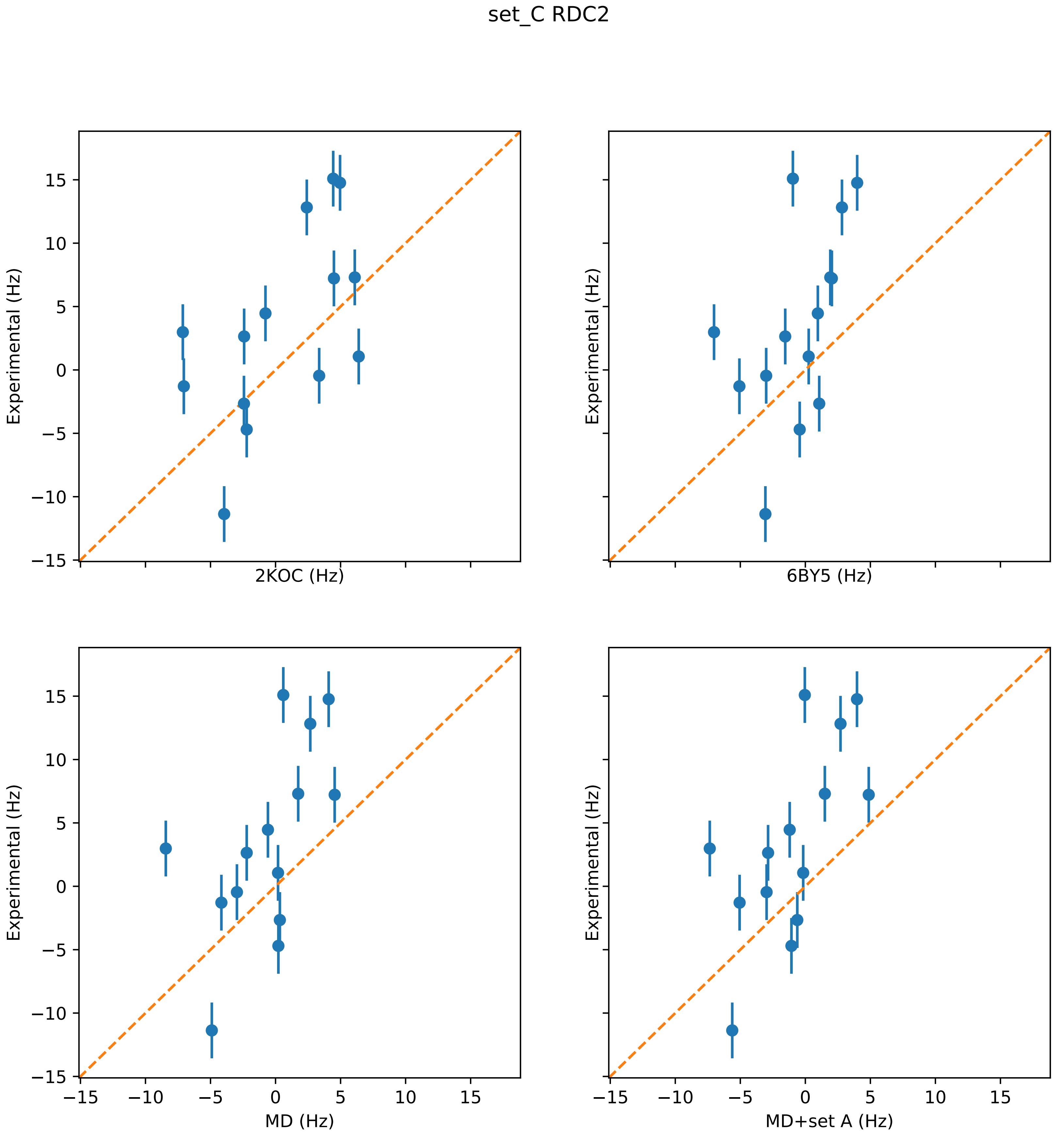
set_C RDC2
Experimental (Hz)
2KOC (Hz)
6BY5 (Hz)
MD (Hz)
MD+set A (Hz)
15
10
5
0
−5
−10
−15
−15
−10
−5
0
5
10
15

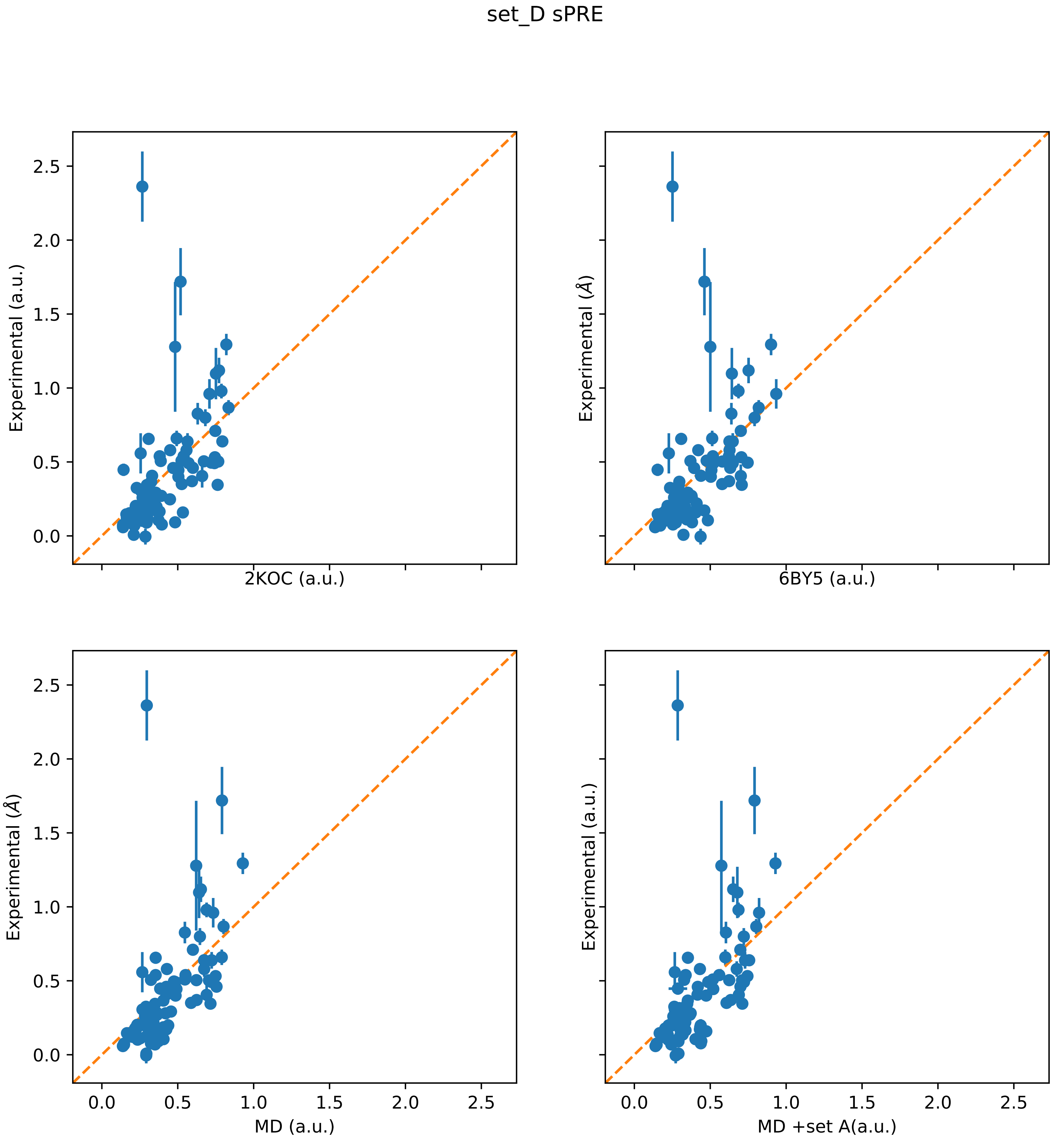

set_D sPRE
Experimental (a.u.)
2KOC (a.u.)
Experimental (Å)
6BY5 (a.u.)
Experimental (Å)
MD (a.u.)
Experimental (a.u.)
MD +set A(a.u.)
0.0
0.5
1.0
1.5
2.0
2.5

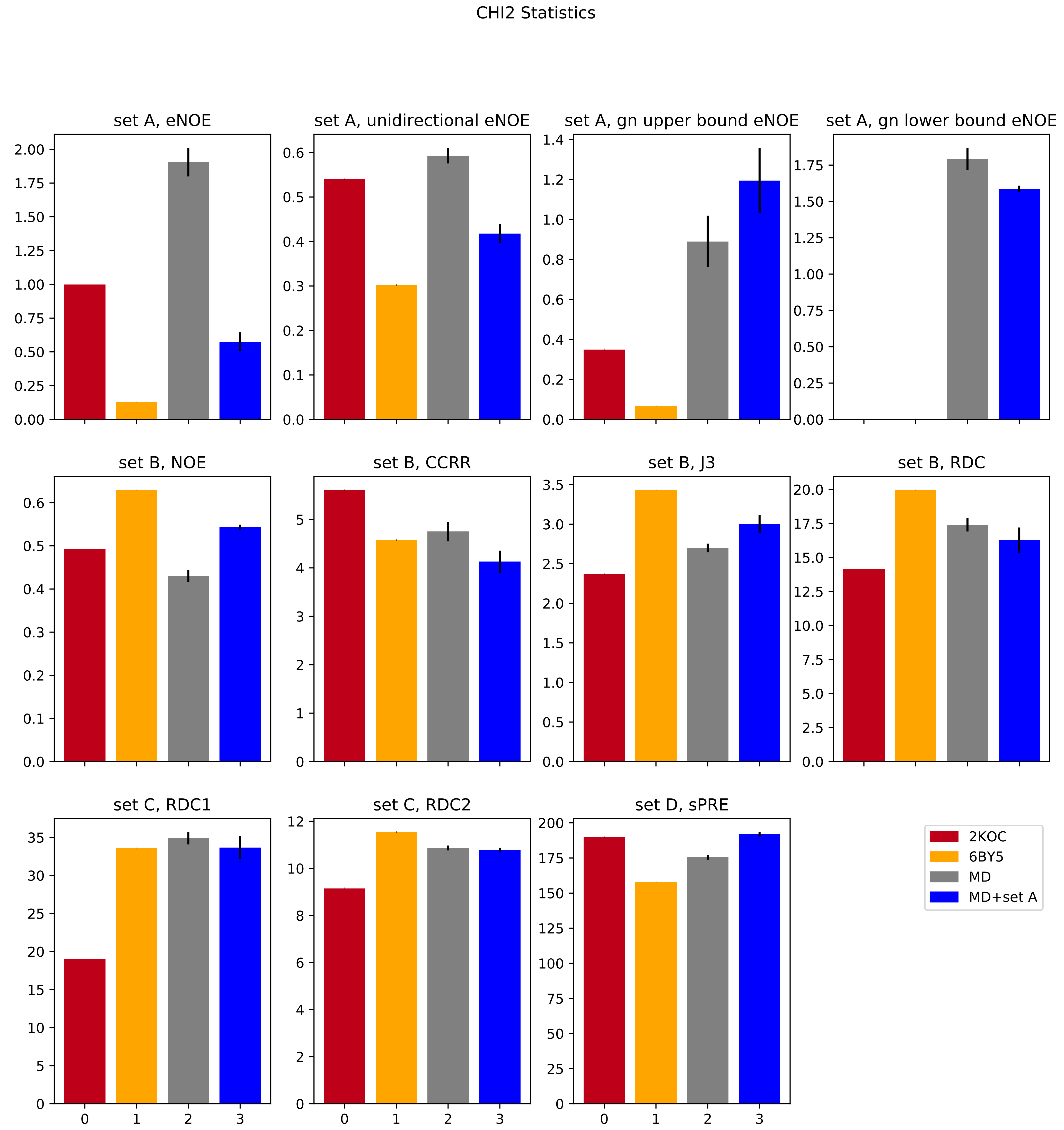
CHI2 Statistics
set A, eNOE
set A, unidirectional eNOE
set A, gn upper bound eNOE
set A, gn lower bound eNOE
set B, NOE
set B, CCRR
set B, J3
set B, RDC
set C, RDC1
set C, RDC2
set D, sPRE
0
1
2
3
2KOC
6BY5
MD
MD+set A

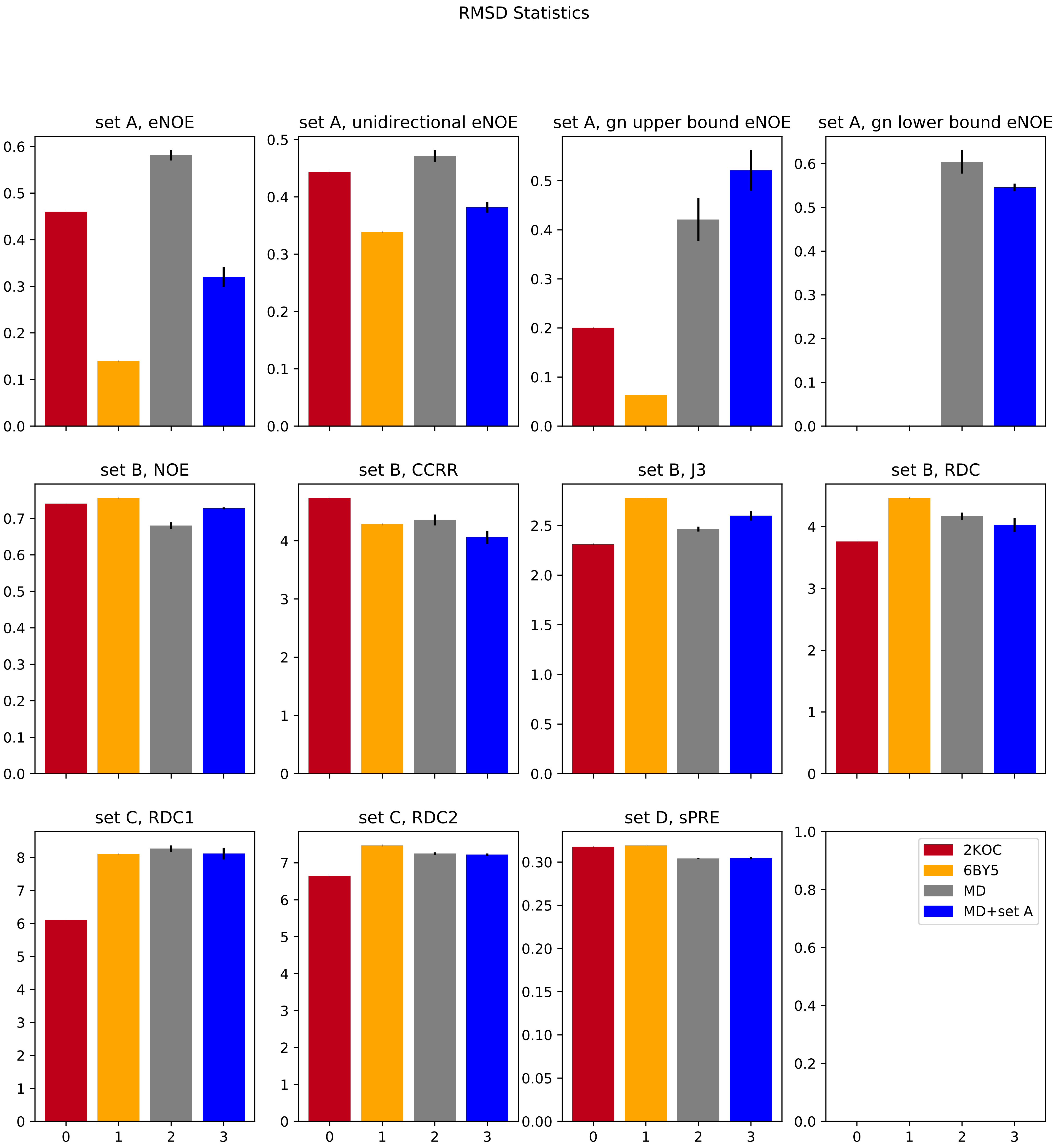

RMSD Statistics
set A, eNOE
set A, unidirectional eNOE
set A, gn upper bound eNOE
set A, gn lower bound eNOE
set B, NOE
set B, CCRR
set B, J3
set B, RDC
set C, RDC1
set C, RDC2
set D, sPRE
2KOC
6BY5
MD
MD+set A
